# The central oxytocinergic system of the prairie vole

**DOI:** 10.1007/s00429-024-02832-1

**Published:** 2024-07-23

**Authors:** E. N. Ramos, G. M. Jiron, J. S. Danoff, Z. Anderson, C. S. Carter, A. M. Perkeybile, J. J. Connelly, A. Erisir

**Affiliations:** Department of Psychology, University of Virginia, Charlottesville, VA USA

**Keywords:** Oxytocin, Electron microscopy, Neuroanatomy, Prairie vole, Light-sheet microscopy, Oxytocin receptor

## Abstract

Oxytocin (OXT) is a peptide hormone and a neuropeptide that regulates various peripheral physiological processes and modulates behavioral responses in the central nervous system. While the humoral release occurs from the axons arriving at the median eminence, the neuropeptide is also released from oxytocinergic cell axons in various brain structures that contain its receptor, and from their dendrites in hypothalamic nuclei and potentially into the cerebrospinal fluid (CSF). Understanding oxytocin’s complex functions requires the knowledge on patterns of oxytocinergic projections in relationship to its receptor (OXTR). This study provides the first comprehensive examination of the oxytocinergic system in the prairie vole (*Microtus ochrogaster*), an animal exhibiting social behaviors that mirror human social behaviors linked to oxytocinergic functioning. Using light and electron microscopy, we characterized the neuroanatomy of the oxytocinergic system in this species. OXT+ cell bodies were found primarily in the hypothalamus, and axons were densest in subcortical regions. Examination of the OXT+ fibers and their relationship to oxytocin receptor transcripts (*Oxtr*) revealed that except for some subcortical structures, the presence of axons was not correlated with the amount of *Oxtr* across the brain. Of particular interest, the cerebral cortex that had high expression of *Oxtr* transcripts contained little to no fibers. Electron microscopy is used to quantify dense cored vesicles (DCV) in OXT+ axons and to identify potential axonal release sites. The ependymal cells that line the ventricles were frequently permissive of DCV-containing OXT+ dendrites reaching the third ventricle. Our results highlight a mechanism in which oxytocin is released directly into the ventricles and circulates throughout the ventricular system, may serve as the primary source for oxytocin that binds to OXTR in the cerebral cortex.

**Supplementary Information:**

The online version contains supplementary material available at 10.1007/s00429-024-02832-1.

## Introduction

The peptide hormone oxytocin has been widely studied for its action in various physiological processes including the initiation of uterine contractions at birth and milk letdown during lactation (Dale 1906; Soloff et al. 1979; Crowley and Armstrong 1992; Perkinson et al. 2021). However, oxytocin as a neuropeptide, also has multifaceted roles in facilitating social bonding, regulating emotional responses and stress, and impacting more complex cognitive functions including aggression, learning, and empathy (Carter 1992; Bale et al. 2001; Bales and Carter 2003; Dabrowska et al. 2011; Olff et al. 2013; Marlin et al. 2015; Jurek and Neumann 2018; Borland et al. 2019; Liu et al. 2020; Giannotti et al. 2022). These behavioral outcomes imply that centrally available oxytocin should have direct effects on various cortical and subcortical brain nuclei, including those in the limbic system, reward pathways, and sensory and association cortices. In support of this idea, the axons of oxytocin-producing hypothalamic cells are found scattered across many subcortical structures and a few cortical areas in several mammalian species studied so far (Marlin et al. 2015; Otero-García et al. 2016; Son et al. 2022). The brain nuclei that contain oxytocin-producing cell bodies are considered among the sites where oxytocin signaling is expected because the neuropeptide can also be released from dendrites containing dense cored vesicles (DCV) (Ludwig et al. 2002; Ludwig and Leng 2006). Similarly, the transcript for the oxytocin receptor (OXTR) has been found in many brain regions (Olazábal and Young 2006; Bosch et al. 2016; Duchemin et al. 2017; Newmaster et al. 2020; Inoue et al. 2022; Son et al. 2022), including the cerebral cortex where oxytocinergic fibers are sparse. Therefore, understanding the patterns of oxytocinergic innervation across the brain nuclei harboring its receptor is crucial for studying oxytocin’s function in the central nervous system.

As an alternative to the most extensively studied rat and mouse species, the prairie vole (*Microtus ochrogaster*) has emerged as a valuable animal model for the study of oxytocin due to its behavioral characteristics that closely resemble human behavior such as pair bonding and alloparenting, which heavily rely on oxytocinergic signaling (Carter and Getz 1993; Perkeybile et al. 2015; Bosch et al. 2016; Hiura and Ophir 2018; Carter et al. 2020; Loth and Donaldson 2021). Research on the prairie vole model has focused on the expression and variability of OXTR (Insel and Shapiro 1992; Perkeybile et al. 2015; Hiura and Ophir 2018; Inoue et al. 2022) but the sources of oxytocin that bind to these receptors remain understudied. The current study uses a combination of brightfield, light-sheet, confocal, and electron microscopy to characterize oxytocin projections, their ultrastructural morphology, and the relationship between oxytocin fibers and *Oxtr* transcript expression. Importantly, oxytocin is often released from axons via en passant boutons without an active zone, making the observations of oxytocin release sites difficult to study (Chini et al. 2017; Oti et al. 2021). At the ultrastructural resolution, potential release sites of oxytocin can be identified by high incidences of DCVs, that is, large vesicles that pack oxytocin in the cell body and transport to its release sites (Lemos and Dayanithi 2020; Oti et al. 2021). The current study uses electron microscopy to quantify the presence of DCVs as a proxy for the neuropeptide release. We further define and elucidate the different modes of oxytocin release in the central nervous system of prairie voles.

## Methods

### Animals

A total of 18 adult prairie voles (*Microtus ochrogaster*) between postnatal days 60 to 90 (PND60-PND90) were used. The voles were descendants of wild-caught stock captured near Champaign, Illinois, and bred at Indiana University (8 animals) and the University of Virginia (10 animals). The animals were pair-housed in polycarbonate cages (27 cm x 16 cm x 16 cm) from the time of weaning on PND21. Animals were given food (high-fiber Purina rabbit chow) and water ad libitum, cotton nestlets for nesting material, and were maintained on a 14:10 light:dark cycle. All animals were nulliparous and sexually inexperienced. All procedures involved in generating tissue for the prairie vole atlas, and in analyzing the oxytocin localization and receptor expression were reviewed and approved by the Institutional Animal Care and Use Committees (IACUC) at Indiana University Bloomington and the University of Virginia. The animals from Indiana University were used for light and electron microscopy studies. Three animals from the University of Virginia were used for light and electron microscopy studies, 2 were used for whole brain clearing and light-sheet microscopy, and the remaining 5 were used for RNAscope in situ hybridization.

### Tissue preparation for light and electron microscopy

For light and electron microscopy studies, 5 male and 6 female prairie voles were deeply anesthetized with an overdose of sodium pentobarbital or isofluorane, and transcardially perfused with Tyrode’s solution (137 mm NaCl, 2 mm KCl, 0.9 mm CaCl_2_, 1.2 mm MgCl_2_, 11.9 mm NaHCO_3_, 0.4 mm NaH_2_PO_4_, 5.5 mm glucose, 281 mOsm, pH 7.4) for 1–2 min, followed by 10–15 ml of 4% paraformaldehyde (for light microscopy), or 4% paraformaldehyde and 0.5% glutaraldehyde (for electron microscopy) in 0.1 M phosphate buffer (pH7.4). After 24 h of post-fixation in the same aldehyde solution, the brains were extracted and sectioned coronally on a vibratome at 50 μm and collected in four series. The second and fourth series were mounted on glass slides and stained for Myelin and Nissl, respectively. The first and third series were rinsed in 1% sodium borohydride and stored in 0.05% sodium azide in 0.01 M PBS at 4 °C until immunostaining experiments.

### Tissue preparation for RNAScope

Five female prairie voles were anesthetized with an overdose of sodium pentobarbital. The brains were immediately extracted on dry ice and stored at –80 °C until sectioning. For cryostat sectioning, the brains were acclimated at –20 °C for at least 2 h, sliced at 15 μm thickness, and mounted onto Superfrost plus slides (Fisherbrand; Pittsburg, Pennsylvania), and stored in slide boxes at –80 °C until fluorescent RNAScope staining.

### Histochemistry for myelin and nissl

For myelin visualization, sections that were mounted on gelatine-subbed slides were rehydrated in 0.02 M PBS for 2 min and incubated in 0.2% HAuCl_4_ for 10–15 min at 60 °C, following a modified myelin staining protocol (Corson et al. 2012). After fine myelination was differentiated, slides were then transferred into an intensification solution of 0.2% KAuCl_4_ for 2–3 min at 60 °C, followed by two rinses in 0.02 M PBS for 2 min each. Sections were incubated in sodium thiosulfate for 3 min and rinsed three times in 0.02 M PBS. Slides were air-dried overnight, dehydrated through a series of ETOH, delipidated in xylenes, and coverslipped with DPX mounting media.

For Nissl visualization, sections were mounted on subbed slides and airdried. The sections were then rehydrated in 100%, 90%, and 70% EtOH solutions, followed by 0.5% cresyl violet in dH2O with 0.3% acetic acid. Slides were then dehydrated in a series of ETOH and xylenes, and coverslipped with DPX mounting media.

### Immunohistochemistry for Oxytocin

For visualization of oxytocin, the sections were pre-incubated for 30 min in 1% BSA, 0.1% Triton-X, 1% BSA in 0.01 M PB, and then transferred to primary antibody rabbit anti-oxytocin (Millipore, CAT#AB911) at 1:20,000 dilution in 0.1 M PBS containing 1% BSA, 0.05% sodium azide and 0.3% TritonX (for light microscopy) for 48 h at room temperature on a shaker Triton-X was omitted from the antibody incubation buffer for electron microscopy experiments. All sections were then rinsed in 0.01 M PBS and transferred to anti-rabbit secondary antibody conjugated to biotin for 2 h, followed by treatment with avidin–biotin-complex (ABC; Vector) solution for 2 h. The sections were then rinsed with 0.01 M PBS and incubated in a solution of 0.02% hydrogen peroxide and 0.05% diaminobenzidine for 2–5 min. The sections prepared for light microscopy were mounted onto subbed slides and coverslipped with DPX mounting media; the other series embedded for electron microscopy.

For pre-embedding gold enhancement staining of OXT fibers, sections were first pre-incubated for 2 h in a solution of 5% BSA in 0.01 M PBS and 0.05% Triton-X, and then incubated in anti-oxytocin antibody, as described above. The sections were then rinsed in 0.01 M PBS and incubated in anti-rabbit IgG conjugated to 1.4 nm gold particles (1:100) in 1% BSA in 0.01 M PBS for 2 h. The tissue was then rinsed in PBS and post-fixed in 1% glutaraldehyde in PBS for 45 min, followed by rinses with 1% BSA in 0.01 M PBS and distilled water. Sections were then treated in GoldEnhance EM enhancement reagent (Nanoprobes, Yaphank, NY) up to 10 min until fiber labeling was detectable by eye. Finally, the sections were rinsed in distilled water, and embedded for electron microscopy as described below.

### Electron microscopy tissue preparation

Resin embedding for electron microscopy followed standard protocols. The sections were treated with 1% osmium tetroxide in 0.1 M PB for 1 h. then transferred into 50% EtOH and counterstained with filtered 4% uranyl acetate in 70% alcohol overnight. The sections were dehydrated through a series of ethanol and acetone solutions and infiltrated with EMbed 812 resin (EMS, Hatfield, PA) overnight. They were then flat-embedded between Aclar sheets (EMS, Hatfield), and placed in a 60 °C oven overnight. Sections containing areas of interest (such as the PVH, SON, NAcc, etc.) were excised and placed in BEEM capsules (EMS, Hatfield). The capsules were filled with resin and were cured in the 60 °C oven overnight, or until polymerized. Areas of interest were traced with a camera lucida and trimmed down to a 1 mm × 2 mm trapezoid-shaped blockface containing labeled neurons or axons. Ultrathin sections (~ 60 nm thin) were cut and collected on 200 mesh copper grids (Ted Pella, Redding, CA) using an ultramicrotome (Ultracut UCT7; Leica, Buffalo Grove, IL). Ultrathin sections of tissue that were visualized with gold-enhanced were counterstained with uranyl acetate, or UranyLess (EMS, Hatfield, PA) and lead nitrate.

### Fluorescent RNAScope for visualizing Oxtr transcripts

Slides containing 2 coronal sections of the forebrain, including nucleus accumbens (Acb) and the anterior cingulate cortex (Cg), and directly posterior to the genu of the corpus callosum were selected for RNAScope in situ hybridization for the mapping of *Oxtr* transcripts. These regions were selected based on previous literature indicating they are regions high in OXTR expression (Inoue et al. 2022). An RNAScope Multiplex Fluorescent Reagent Kit version 2 (Advanced Cell Diagnostics; Newark, California) was used for fluorescent staining of *Oxtr* transcripts, according to the manufacturer’s instructions. Briefly, slides were fixed in 4% paraformaldehyde in 4 °C for 1 h, and then dehydrated through a series of ethanol solutions of 50%, 70%, and two 100%, 5 min each. The slides were then stored for up to one week at −20 °C. On day 2 of staining, the slides were blocked in hydrogen peroxide, incubated in protease reagents, the *Oxtr* probe (CAT. No. 500721), and three amplification reagents. The hybridized probes were visualized using OPAL 690 and counterstained using DAPI. Finally, slides were coverslipped with ProLong Gold Antifade Mountant (ThermoFisher Scientific; Waltham, Massachusetts). A negative, bacterial DapB (Cat. No. 320871), and a positive, PPIB (Cat. No. 533491) control probes were run in parallel. For the negative control, there was no specific labeling, and for the positive, there were  > 15 puncta per cell.

### Whole-brain tissue clearance using CLARITY and light-sheet microscopy

Two prairie vole brains (1 male, 1 female) were cleared using the CLARITY clearance method. The animals were perfused as described above and post-fixed for 24 h. Then, the whole-brain samples were preserved using SHIELD reagent (LifeCanvas Technologies & Folorunso et al., 2023). Brains then cleared using the delipidation buffer for 24 h followed by SmartBatch + Delipidation for 30 h (LifeCanvas Technologies). For immunolabeling, the cleared brains were incubated in the primary antibody, anti-rabbit OXT, in 5% normal donkey serum (1:5000) for 18 h. Samples were then washed and fixed in 4% PFA overnight and treated with fluorescent-conjugated anti-rabbit secondary antibody (donkey anti-Rabbit IgG Alexa Fluor 488; Invitrogen, CAT#A32790; 1: 200).

### Imaging and Construction of the Prairie Vole Atlas

**Fig. 1:**
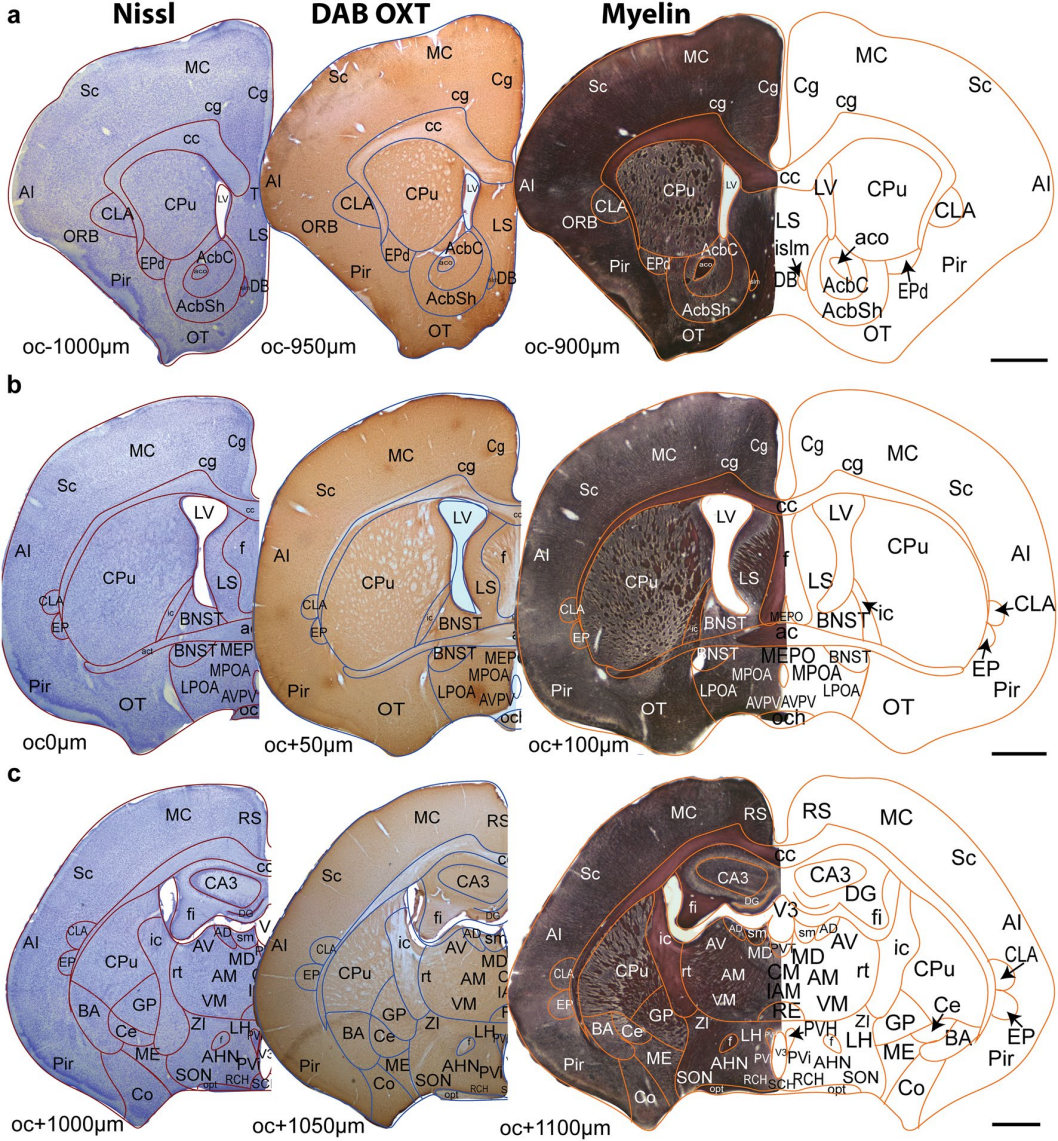
Adjacent coronal sections that are 50 μm apart were stained with Nissl, Oxytocin, or Myelin, and annotated to create the prairie vole brain atlas **A-C** Three series that are 1000 μm apart are illustrated. The anterior-most section of the optic chiasm **B** is denoted as the oc0 coordinate, and the sections anterior and posterior to oc0 are marked as oc− and oc+, respectively. Scale bar = 1 mm

To construct an annotated atlas of the prairie vole brain as an open resource tool, all Nissl, Myelin and oxytocin-stained series from a male prairie vole brain were imaged using a Leica MC170 HD microscope at 1.6 × and 20 × magnifications. The higher magnification images of Nissl or Myelin-stained sections were tiled to make composites of each brain section, using Adobe Photoshop. The Adobe Illustrator software was used to make aligned sets of Nissl, Myelin and oxytocin labeled sections, representing 200 µm thick coronal planes. The Nissl images were used to outline the section boundaries and the cytoarchitectural borders of all major brain nuclei. These outlines were superimposed on the images of the myelin-stained section in the set, and the outlines of myelinated axon bundles and other differentiated structures were added (Supp Fig. 1). Demarcated brain regions were annotated using the terminology adopted in the Prairie Vole MRI Brain Atlas (Yee et al. 2016), the Allen Mouse Brain Atlas (Allen Institute for Brain Science, 2004), or the Paxinos and Watson Rat Brain Atlas (Paxinos and Watson 2007). Thirty-three series extending from the olfactory bulb to the pons were compiled in an anterior–posterior coordinate plane using the anterior-most coronal section that contained the optic chiasm (oc) as the AP zero coordinate. All sections anterior and posterior to the oc are designated as ‘oc−’ and ‘oc+’, respectively (Fig. 1A–C). A pdf version of the prairie vole atlas (The Histochemical Brain Atlas of The Prairie Vole (*Microtus ochrogaster)*) is accessible at 10.18130/V3/LSAONY.

### Imaging and analysis of OXT+ cells and fibers

For mapping the location of oxytocinergic cells and the fibers on the atlas, we examined all OXT-stained sections (every other 50 µm coronal section) of the brain used for the Atlas on the Zeiss Axio Imager M2 microscope with 20 × and 40 × objectives. When an OXT+ fiber or cell was encountered, we switched to a low magnification objective to identify its location in reference to the Prairie Vole Brain Atlas outlines drawn on adjacent Nissl and Myelin sections. Every fourth coronal section from 5 other brains (2 males and 3 females) was also examined using the same strategy to map the location of cells and axons.

All encounters of labeled cell bodies within an identified brain region were evaluated to allocate a semi-quantitative staining density score: regions with 0 cells received a score of 0; 1-6 cells received a score of 1; 6-12 cells received a score of 2; and 12 or more cells received a score of 3. The same staining density quantification strategy was used to map the locations of OXT+ axons: a score of 0 was given when no axon was encountered in the section; + for 1 axon in a small region or 2-4 axons in a large cross-section of a region, such as caudate/putamen; ++ for when neither  + or +++  criteria was met; and +++ for when the fibers are unquantifiably dense, such as in lateral septum, or completely filling a small region, such as in s. nigra.

Because staining density scores varied across sections that contain a brain region, and sparsely labeled regions may display no fibers or cells on some sections, it was important to normalize the staining densities to the size of each brain region. For that purpose, a Staining Density Index (SDI) was calculated by using the formula (3N3 + 2N2 + N1)/3NT, where N3, N2, and N1 are the number of sections with a  +++, ++, or + score, respectively, and NT is the number of sections the region spans, including the sections with no fibers or cells.

### Imaging and analysis of oxtr transcripts via RNAScope

For RNAScope imaging of *Oxtr* transcripts, a STELLARIS 5 (Leica Microsystems) confocal microscope with a 10 × objective was used for whole section fluorescence imaging, and a 40 × objective for high-magnification z-stack imaging of sections containing the Acb and Cg. Identical settings of the gain and intensity were used for each brain and image.

A publicly available comprehensive dataset of *Oxtr* transcripts in prairie vole brain (Inoue et al. 2022) was used for comparing with our OXT+ fiber quantification data. This dataset is a semi-quantitative assessment of the quantity of *Oxtr* transcripts across the entire prairie vole brain and provides a score between 1 and 4 for each brain nuclei. To align the two datasets, the lists of brain regions included in the two studies are compared, and discrepancies are noted. If a region in *Oxtr* dataset had more subregions than our OXT fiber dataset, such as the BNST, the *Oxtr* score was averaged across subregions to yield one score. Any region included in our dataset that could not be matched to the *Oxtr* regions was excluded. The regions that were in the OXT dataset yet no corresponding *Oxtr* score was provided were also excluded. For a full list of regions included in the OXT/*Oxtr* comparison analysis, see Supplemental Table 3. All regions were further categorized into the thalamus, hypothalamus, midbrain, striatum/pallidum, and cerebral cortex, based on the classification of these regions within the Allen Mouse Brain Atlas. For a full list of regions included in the OXT/*Oxtr* comparison analysis, as well as their classification, pooled OXT SDI scores from all female and all male brains, and *Oxtr* scores, see Supplemental Table 3. OXT SDI scores were pooled because subsequent analysis revealed few sex differences. Data were analyzed using a linear regression model and the lm function in R, and further analyzed through the emmeans package and emtrends function in R to determine estimated linear trends of each subregion.

### Imaging and analysis of OXT+ neuropil and DCVs via electron microscopy

Electron microscopy images were captured on a JEOL 1010 EM with a 16-megapixel CCD camera (SIA) at 15,000X magnification, yielding 0.8 nm/pixel resolution. Cross-sections of dendrites were identified by the presence of microtubules and lack of neurotransmitter vesicles. The presence of myelin sheets is an identifier for myelinated axons. Unmyelinated axons display uniform calibers in longitudinal cross-sections, neurofilaments and fewer microtubules, and they do not receive synapses. Axon terminals may display presynaptic zones, and contain vesicles, including dense cored vesicles. DCVs are membrane-bound round structures with dark centers. DCVs can be in various sizes but they are larger than neurotransmitter vesicles, which have clear centers. We used the presence of DCVs as a proxy for OXT release. Because OXT is not the only peptide packaged into DCVs, nor is OXT the only molecule present in OXT+  cells, we characterized OXT+ DCVs by their unique sizes (Makani et al. 2013). To determine the prototypical size of DCVs in OXT+  and OXT- profiles, we examined OXT+  cell bodies within the PVH and unstained (OXT−) regions displaying DCV’s, used the freehand selection tool in ImageJ to draw borders along all DCVs, and measured their areas. This analysis revealed size differences between OXT+ and OXT− DCVs and yielded a cutoff size criterion for OXT+ DCVs. The cutoff size was determined as the intersection of the density plots between OXT+ DCV’s and OXT− DCV's, where the density of OXT+ DCVs falling below the cutoff value was less that 5% of the measured OXT+ DCV’s.

### Imaging and analysis of whole-brain clarity tissue

For 3D imaging of cleared and OXT-stained brains, a Zeiss Lightsheet 7 microscope at 5X magnification was used. Whole-brain 3D images were examined using Imaris Imaging Software.

All statistical analysis was done using R Studio and the ggplot package for data visualization. For comparisons of labeling density across animals, Welch’s t-test was used. *Oxtr* vs OXT+ fiber density data were analyzed using a linear regression model and the lm function in R, and further analyzed through the emmeans package and emtrends function in R to determine estimated linear trends of each sub-region. Graphs were made on R Studio. Adobe Illustrator and Adobe Photoshop were used for constructing all figures.

## Results

### Prairie vole atlas

As we aimed to quantify the oxytocin-stained neuronal elements within identified brain nuclei in the prairie vole model, we started with composing an annotated brain atlas of the prairie vole based on serial coronal sections of a male animal brain, stained for visualization of cytoarchitecture and myelinated fiber tracks (See Methods). The current pdf version of the atlas, The Histochemical Brain Atlas of The Prairie Vole (*Microtus ochrogaster),* released with this manuscript (10.18130/V3/LSAONY) contains 33 annotated brain atlas templates, representing 200 µm thick coronal plates (Fig. 1). Each plate includes superimposed images of Nissl and Myelin sections that were used for demarcating and annotating brain regions on templates. The plates also contain oxytocin immuno-stained sections that we used for fiber and cell mapping analysis, as described below. Annotated templates are provided as vector images, which can be copied by future users on their own material (Ramos and Erisir 2024).

### Brain regions containing OXT+ cell bodies and dendrites

**Fig. 2:**
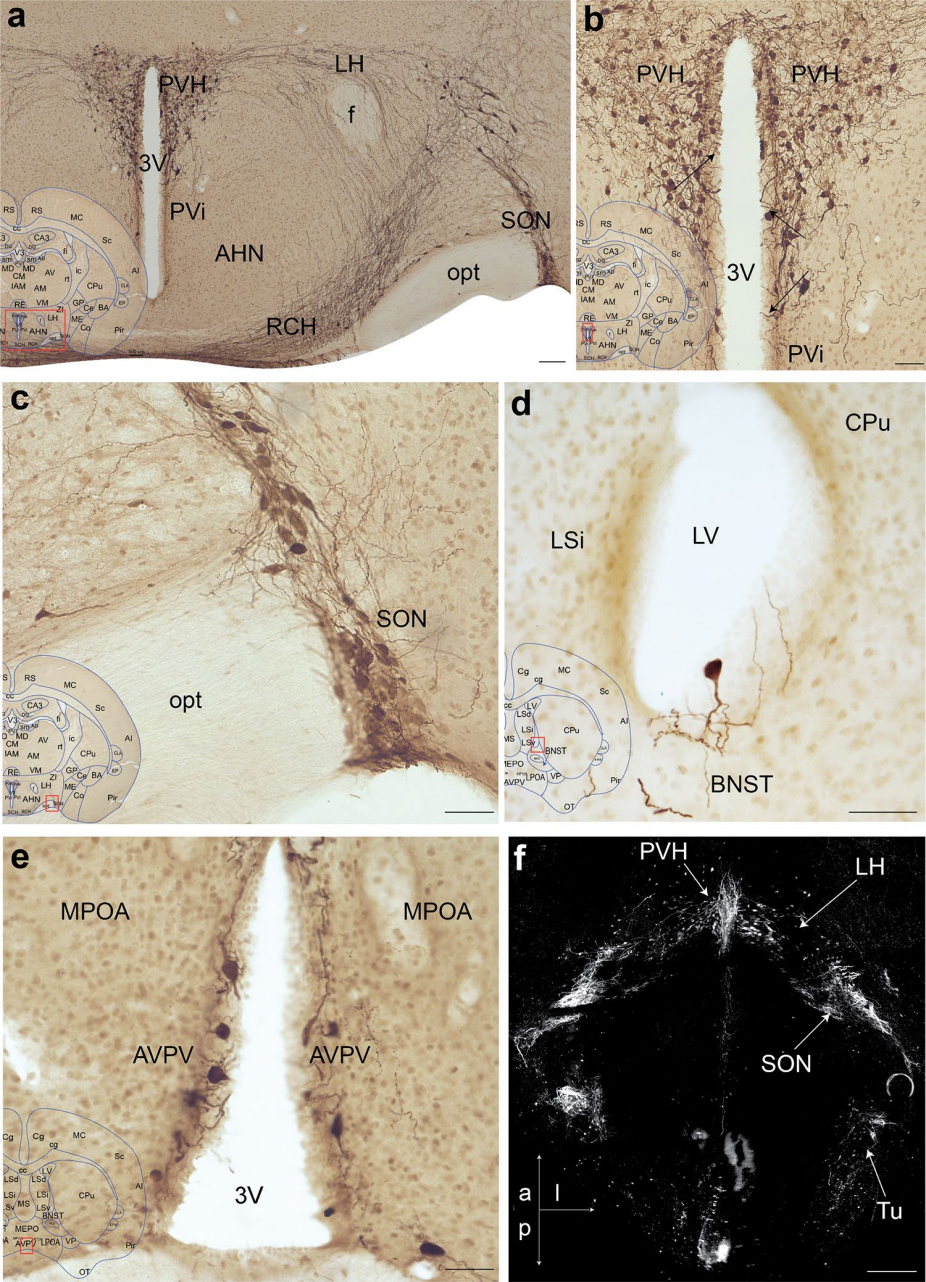
OXT+ cell bodies in prairie vole brain primarily reside in the hypothalamus. **A** An OXT-stained coronal section through the hypothalamus reveals darkly stained cell bodies in the PVH, SON, and LH. The superimposed inset marks the region on a corresponding atlas section in red and applies to panels A to E. Scale bar = 100 μm. **B** Upon higher magnification, the OXT+ PVH cell bodies are multipolar. Those that are close to the ventricle extend dendrites into the ventricular lining (black arrows). Scale bar in B = 500 μm and applies to panels B to E. **C)** In the SON, OXT+ somata are fusiform and multipolar, and project dendrites toward the ventral surface of the brain. **D** Ventricular lining surrounding lateral ventricles occasionally displayed solitary OXT+ somata, which extend processes toward the ventricle. These are referred to as ectopic cells in the text as they cannot be identified as belonging to any brain nuclei. The example in panel D is close to BNST, the only extra-hypothalamic region that consistently contains OXT+ cells. **E** The presence of OXT labeling in AVPV cells is unique to the prairie vole. **F** Light-sheet microscope image of the hypothalamus at 5 × objective in the horizontal plane, thick white arrows illustrate cell body groups within the PVH, LH, SON, and Tu, thin white arrows in the lower left corner indicate anterior (a), posterior (p), and lateral (l) directionality of the image, maximum intensity projection across 1100 μm, scale bar = 500 μm

We used The Histochemical Atlas of Prairie Vole Brain as a reference template to map the locations of oxytocin-producing cell bodies in the prairie vole brain. In DAB-labeled sections, oxytocin immunostain (OXT+) fills the somata in their entirety, as well as the dendrites emanating from the soma (Fig. 2A-E). Most cells have a multi-polar structure, with dendrites extending in different directions. Semi-quantitative scoring and computation of a Staining Density Index (see Methods) for each region allowed assessments of the prevalence of OXT+ cells.

The hypothalamic regions had the highest density of OXT+ cells (Fig. 2A & F). The OXT+ cells are densest in the PVH and SON (Fig. 2B, C), followed by the tuberal nucleus (Tu) and periventricular hypothalamic preoptic part (PVpo). OXT+ cells are sparser in other regions, including the lateral hypothalamus (LH) and medial preoptic area (MPOA). An extrahypothalamic region, the BNST, consistently contains OXT+ cell bodies that closely line the ventricle and occasionally appear ectopic, and as such, do not belong to one particular brain region (Fig. 2D). Dendrites of cells in PVH, SON, and BNST often are located close to and sometimes within the ventricular lining (Fig. 2B–D). As described before (Kelly et al. 2018; Kenkel et al. 2021), the distinction between magnocellular and parvocellular cells that are typically seen in other rodent models, such as mice and rats, is not as apparent in the prairie vole brain. Although the rostral part of the PVH primarily encompasses the magnocellular cell group and the caudal part encompasses the parvocellular group of the prairie vole, no differences were seen within the cell morphology of the PVH. For the full list of cell body locations see Supplemental Table 1. Finally, light-sheet microscopy corroborated that the densest cell body localizations reside in the hypothalamus (Fig. 2F).

To reveal potential sexual dimorphisms, cell body SDI scores from 3 male and 3 female vole brains were compared using Welch’s t-tests. No statistical differences were found (Supplemental Fig. 2a), suggesting that sexual dimorphisms seen in behaviors modulated by OXT cannot be explained by the localization or the density of oxytocinergic cells.

To address potential species differences, we compared the localization of cells in each brain region of the prairie vole to previous studies in the mouse, hamster, and rat (Swaab et al. 1975; Laurent et al. 1989; Whitman and Albers 1998; Otero-García et al. 2016; Son et al. 2022). While most OXT+ cell localizations are similar, we noted two differences. First, similar to hamsters and unlike other rodents, the prairie vole has OXT+ cells within the median preoptic nucleus (MEPO) (Whitman and Albers 1998). Second, the prairie vole has a group of OXT+ cells located in the anteroventral periventricular nucleus (AVPV), which has not been noted in any other species (Fig. 2E). Interestingly, AVPV is among the regions in which processes project towards the ventricle and occasionally through the ependymal cell layer in the prairie vole (Fig. 2E).

### Brain regions containing OXT-labeled axons

**Fig. 3:**
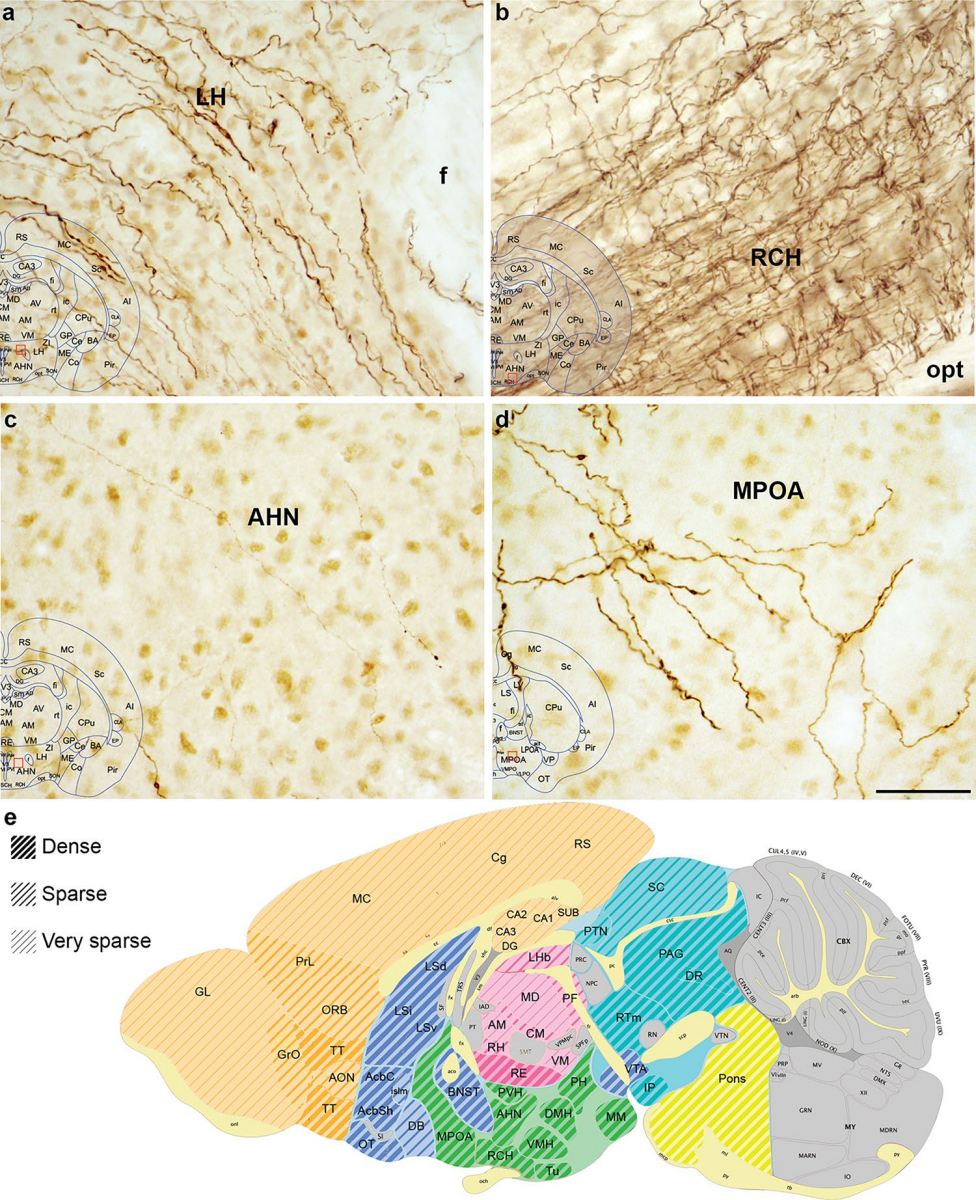
Locations of OXT+ fibers within the prairie vole LH (**A**), RCH (**B**), AHN (**C**), and MPOA (**D**). The superimposed inset marks the region on a corresponding atlas section in red. Scale bar = 50 μm and applies to panels A to D.** E**) Illustration of fiber density data (Supplemental Table 3) over a sagittal section adapted from Allen Mouse Brain Atlas (mouse.brainmap.org). The density of fibers (Supplemental Table 3) is represented in tertiles (dense, sparse, and very sparse, which was determined within the cerebral cortex (orange), hindbrain (yellow), hypothalamus (green), striatum/pallidum (blue), midbrain (teal), and thalamus (pink). See Supplemental Table 3 for full list of brain regions, their SDIs, and their classifications into the subregions (cerebral cortex, hindbrain, etc.),

Next, we used DAB-labeling of OXT with brightfield microscopy to examine all major nuclei containing OXT axonal projections. On coronal sections, two distinct projection paths emerging from the PVH can be observed: one consists of a dense bundle of axons that courses dorso-laterally, and another that contains sparse and thin axonal fibers that project more ventro-medially (Fig. 2A). The dorso-lateral axons extend horizontally above the anterior hypothalamic nucleus (AHN) and make a sharp ventral turn at the lateral hypothalamus (Fig. 2A, Fig. 3A). PVH axons seem to merge with another stream of axons emerging from the SON and these course along the ventral surface of the brain along the retrochiasmatic nucleus (RCH) and above the optic tract (opt) towards the median eminence (ME) (Fig. 3B). The axons of this dorso-lateral stream are thick and display swellings throughout their course (Fig. 3A).

The ventro-medial axons emerging from the PVH follow a less defined course, winding throughout the AHN (Fig. 3C). These axons do not appear to be joining the OXT axon stream projecting towards the median eminence and are very fine displaying many varicosities (Fig. 3C). Further, there are axons in other regions of the hypothalamus, anterior and posterior to the appearance of the PVH and SON, which show thick fibers with large swellings such as the MPOA (Fig. 3D).

The OXT+ axons are dense in many non-hypothalamic subcortical brain regions, including the periaqueductal gray (PAG), ventral tegmental area (VTA), pons, nucleus reuniens of the thalamus (RE), BNST, and AcbSh and AcbC (Fig. 3E. Note: classifications into “dense”, “sparse”, and “very sparse” were made based on tertiles that can be derived from the pooled data in Supplemental Table 3. Dense regions: SDIs = 0.46–1; sparse regions: SDIs = 0.07–0.42; very sparse regions: SDIs = 0–0.05). Various sub-cortical regions appeared to have dense fiber staining, while labeled axons were extremely rare in cortical regions. Interestingly, OXT+ axons were observed touching all major ventricles and were prominent in many areas that border the ventricles, such as the lateral septum (LS), BNST, paraventricular nucleus of the thalamus (PVT), and PAG (Fig. 3E; for the full list of axonal locations and the SDIs of individual subjects see Supplemental Table 2; for all axonal locations and the pooled SDIs see Supplemental Table 3).

Sexual dimorphisms for OXT+ fibers were examined across 105 brain regions in 3 male and 3 female prairie vole brains. Regions that only had one score in either males or females were excluded because a mean value could not be calculated from one observation. While our sample size was not sufficient for statistical power in population comparisons, comparison of SDIs within each brain region revealed two regions that may be sexually dimorphic: the dorsal endopiriform cortex (EPd), and the prelimbic cortex (PrL) (Supplemental Fig. 2b). Unlike in male brains, female brains did not display any fibers in Epd *(*0.17 ± 0.026 vs. 0 ± 0; mean ± SD; Welch’s t-test, t(2) = -11.7, p = 0.007)) Males also had higher OXT fiber density in the PrL than females (0.25 ± 0.07 vs. 0.04 ± 0.06; Welch’s t-test, t(3.95) = −3.88, p = 0.02; Supplemental Fig. 2b). These results suggest that perhaps males utilize more OXT in the EPd and PrL than females, and this may play a role in downstream behavioral outcomes.

### Comparison of brain regions with OXT+ fibers and Oxtr transcripts

**Fig. 4:**
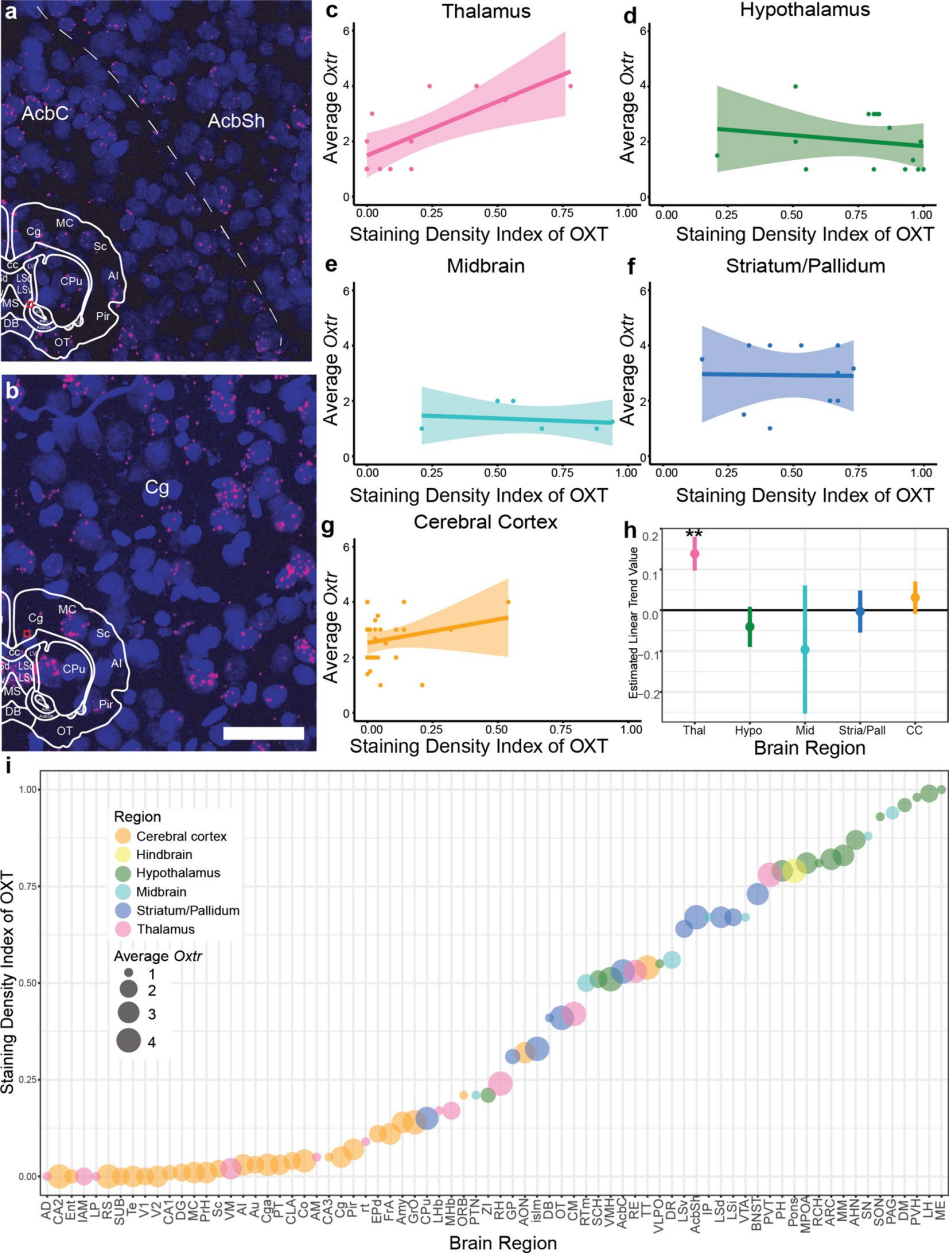
Correlation of OXT+ fibers and *Oxtr* across the whole brain. **A** Confocal images of *Oxtr* transcript expression (pink) within the AcbSh and AcbC (**A**), and the Cg (**B)**. Cell nuclei are stained with DAPI (blue). Scale bar = 50 μm. **C-G** Graphs shown are illustrations of the raw data (brain regions belonging to category are shown as points), regression lines (solid lines) and 95% confidence intervals (shaded areas around regression lines), these are shown for visualization purposes, see H for full statistical analyses. **C** The density of OXT+ fibers within the thalamus is positively related to density of *Oxtr* transcript (*p* = 0.001), but not in hypothalamus (**D**, *p* = 0.41), midbrain (**E**, *p* = 0.54), striatum/pallidum (**F**, *p* = 0.95), or the cerebral cortex (**G**, *p* = 0.44). **H**) Graphical representation of estimated linear trend analysis illustrating that the thalamus (Thal, pink) is the only region where presence of OXT+ fibers predicts *Oxtr*. The hypothalamus (Hypo, green) and midbrain (Mid, teal) both have negative, non-significant trend values with a range of OXT+ fibers (often high) and little *Oxtr.* The striatum/pallidum (Stria/Pall, blue) has a trend value of near 0, thus no relationship between OXT+ fibers and *Oxtr*. The cerebral cortex (CC, orange) has a positive, nonsignificant trend value, indicating typically low OXT+ fibers and varying amounts *Oxtr* (often high). **I** The staining density index (see Methods) at all regions in descending order from the highest density of OXT+ fibers (top) to the lowest density of OXT+ fibers (bottom) colored by region category. The relative amount of *Oxtr* is indicated by the size of the circle marker

To confirm that the animals used in the current study displayed *Oxtr* expression patterns similar to that were recently described (Inoue et al. 2022), we examined sample brain regions that were reported to express high levels of *Oxtr*, yet show vast differences in their OXT+ fiber density in our study. In particular, we examined the *Oxtr* prevalence in the Nucleus Accumbens Shell (AcbSh), Core (AcbC), and Cingulate Cortex (Cg). Although both regions reportedly have high *Oxtr* levels (Fig. 4A-B), fiber density analysis yields different SDI values between the Acb (pooled SDI Sh: 0.67 and C: 0.53) and Cg (0.05) (SuppTable3). The RNAScope visualization of *Oxtr* RNA transcripts (*Oxtr*) in 10 sections from 5 animals revealed that both the Acb (Sh and C) and Cg contain an abundance of *Oxtr-*expressing cells (Fig. 4A, B). Specifically, 15.4% ± 6.9 of cells in AcbSh, 23.9% ± 13.1 of cells in AcbC, and in the Cg 18.4% ± 5.3 of cells in Cg expressed *Oxtr.* The puncta density (i.e. counts of *Oxtr* puncta per cell) was 3.6 ± 1.6 in AcbSh, 3.4 ± 1.8 in AcbC, and 8.4 ± 2.2 in Cg. These results confirm that the Acb and Cg cells both highly expressed *Oxtr* in our sample brains, comparable to the Inoue et al. 2022*Oxtr* dataset, where the Cg received a score of 3, and the Acb (Sh and C) received scores of 4 (Fig. 4A, B; Supplemental Table3).

In order to analyze the relationship between OXT+ fiber density and *Oxtr* localization across the prairie vole brain, we compared our OXT+ fiber SDI data with a dataset of previously published quantitative distribution of *Oxtr* transcripts (Inoue et al. 2022) (Fig. 4C–I). We examined this relationship across 5 parent regions as established by the Allen Mouse Brian Atlas (mouse.brainmap.org): the cerebral cortex, hypothalamus, midbrain, striatum, and pallidum, and the thalamus (for brain regions included in each category, see Suppl. Table 3). This analysis revealed a significant interaction effect between *Oxtr* and brain region, suggesting that the relationship between OXT+ fibers and *Oxtr* transcripts differs depending on region (linear regression, F_(1,63)_ = 2.47, *p* = 0.05). Interestingly, the subregions within each parent category tend to cluster together in relation to their OXT fiber density, and they typically have a similar level of average *Oxtr* (Fig. 4I). To further probe the differences amongst regions, we utilized linear trend analysis. There was a general directionality of the trends yet, in most areas, the amount of OXT fibers was not correlated with the amount of *Oxtr.* There was a negative but non-significant relationship in the hypothalamus and midbrain; no relationship in striatum/pallidum, and a non-significant positive relationship in the cerebral cortex (Fig. 4H). Only the thalamus category (which includes RE, PVT and CM) displays a significant relationship between OXT+ fibers and *Oxtr* transcript (estimate linear trends analysis, Trend = 0.13, *p* = 0.001; Fig. 4C). This suggests that within the brain structures included in the thalamus category, the amount of *Oxtr* transcripts can be explained by the amount of OXT+ fibers. That is, the axonal release in the thalamus may constitute a primary source for OXTR protein binding.

In contrast, in the hypothalamus and midbrain categories, there is no significant relationship between OXT+ fibers and *Oxtr* (Trend = −0.04, *p* = 0.41; and Trend = -0.010, *p* = 0.54). While the fiber density in these areas is typically high, *Oxtr* density is low, suggesting that OXT+ fibers in these regions are likely axons of passage (Fig. 4D, E). The striatum and pallidum display no significant relationship between OXT+ fibers and *Oxtr.* These areas typically have high amounts of *Oxtr* yet moderate to high (> 0.15SDI) levels of OXT+ fibers (Trend = -0.003, *p* = 0.95, Fig. 4F). Similarly, the cerebral cortex category contains only a few regions with scant amounts of OXT+ fibers, but many regions display high levels of the *Oxtr* transcript (Trend = 0.03, *p* = 0.44, Fig. 4G). This mismatch in the amount of OXT+ fibers and *Oxtr*, particularly in cortex and striatum/pallidum regions with high *Oxtr* but no or few OXT+ axons, indicates that the receptor localization cannot be explained by the presence of OXT+ fibers alone and that these regions may be getting the bulk of their oxytocin through non-axonal means.

### Ultrastructural characteristics of OXT+ axons in regions with differing amounts of Oxtr transcripts

**Fig. 5:**
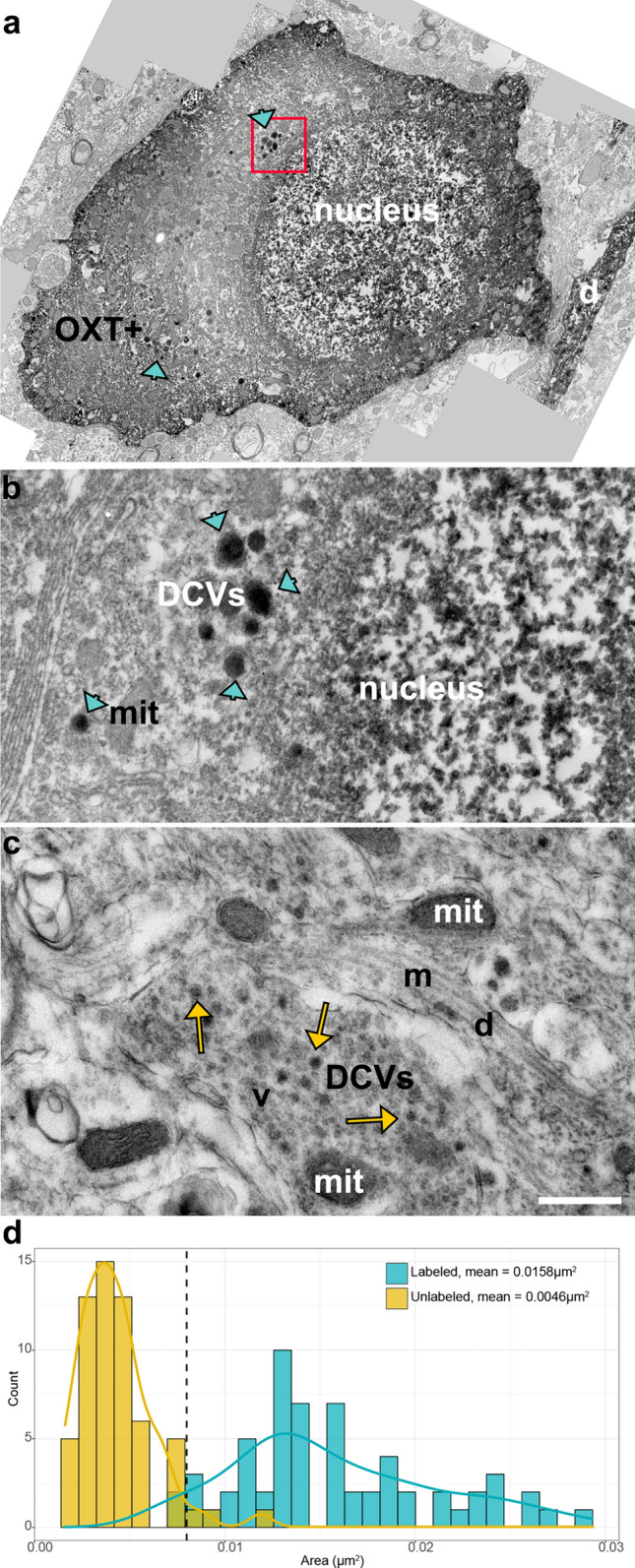
OXT+ somata in PVH **A** display clusters of dense cored vesicles (blue arrowheads, DCVs) in the cytoplasm (**A**,**B**). Unlabeled DCVs (yellow arrows) were also observed in PVH neuropil (**C**). Scale bar = .5 μm and applies to (**B**, **C)**. **D** Frequency distribution histogram of OXT+ (blue bars, n = 61) and OXT− (yellow bars, n = 60) dense core vesicle areas, blue and yellow line overlays represent density curves for labeled and unlabeled distribution respectively. The dashed line marks the cutoff value for classifying DCVs as oxytocinergic. OXT+ : oxytocin positive cell, DCVs: dense cored vesicles, mit: mitochondria, m: microtubules, d: dendrite

**Fig. 6:**
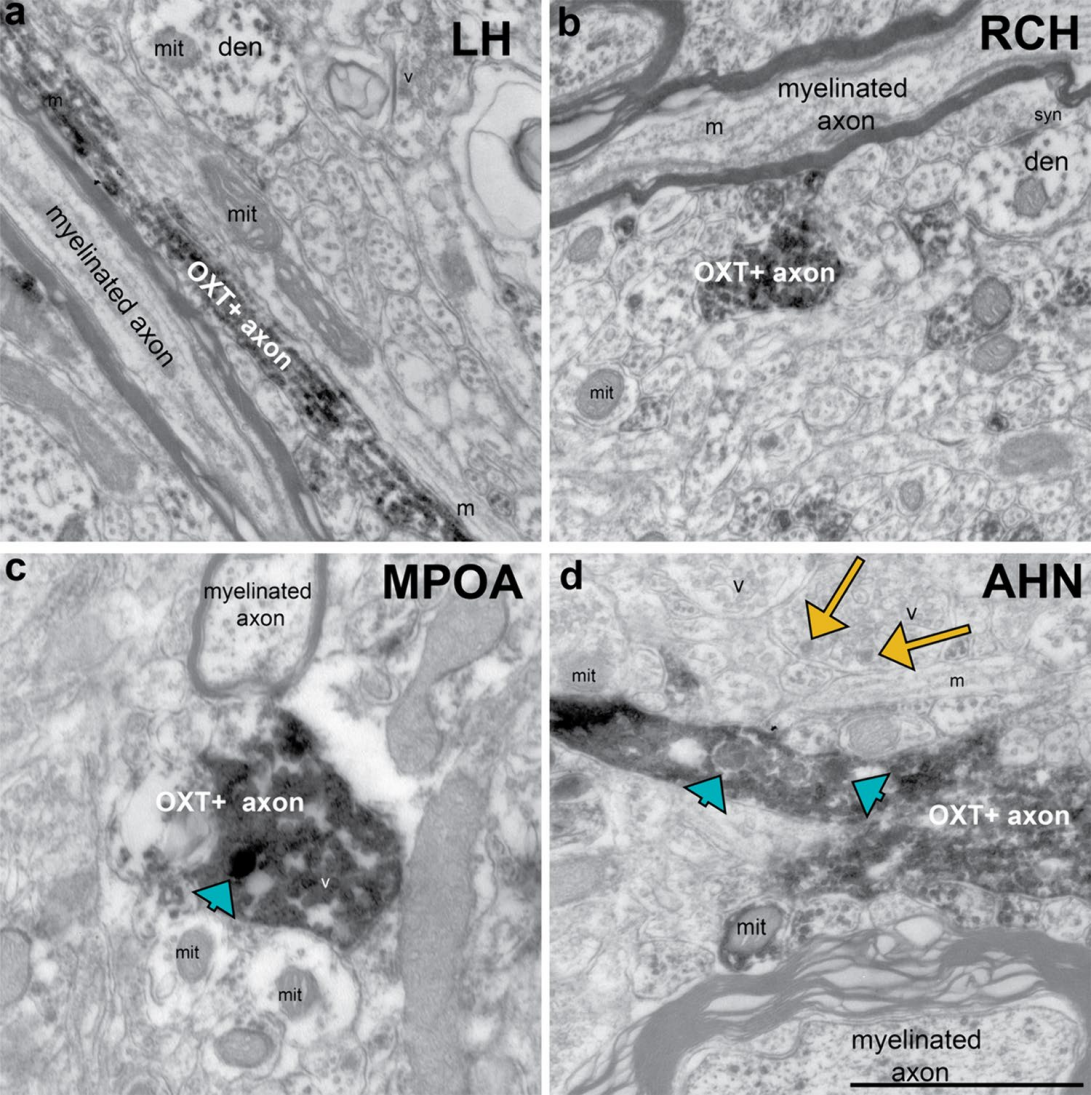
OXT+ axons of the LH and RCH typically do not contain DCVs, indicating they are likely axons of passage, while axons of the MPOA and AHN often contain DCVs. **A** An Electron micrograph of OXT+ axons in the LH, and **B** in the RCH, with no DCVs. **C** EM image of an OXT+ axon in the MPOA with one DCVs (blue arrowhead), and **D** an image of an axon in the AHN with two DCV’s (blue arrowheads), and unstained neuropil with DCV’s (yellow arrows), indicating in the MPOA and AHN oxytocin can be directly released to act on OXTR. Pixel resolution = 1134.92 pixels/μm. Scale bar = 1 μm. Mit: mitochondria, v: vesicles, den: dendrite, m: microtubules, syn: synapse

To characterize the fine morphological properties of OXT+ fibers in regions of high or low levels of *Oxtr*, we examined the LH, RCH, MPOA, and AHN regions immunostained for OXT using transmission electron microscopy. The OXT+ labeling was evident as the appearance of electron-dense DAB chromogen diffusely filling the profiles of neurons, dendrites, and axons (Fig. 5, 6). In regions where diffuse DAB label in profiles was too dense and obscured the organelles within, pre-embedding gold enhanced visualization approach was used, revealing OXT+ profiles with the appearance of irregularly shaped gold deposits (Fig. 6E).

Within OXT+ profiles, darkly stained, large DCVs often appeared in clusters within labeled somata and neuropil (Fig. 5A, B). Because DCVs of various sizes were also observed in unlabeled profiles (Fig. 5C, yellow arrows), we quantified the size of labeled and unlabeled DCVs in the PVH to obtain a size criterion for OXT+ DCVs. The DCVs in labeled PVH cells were uniformly large (0.016μm^2^ ± 0.005, n = 61; ~ 140 nm in diameter) and these could be distinguished from other DCVs encountered in the OXT− neuropil (0.004μm^2^ ± 0.002, n = 60; ~ 70 nm in diameter) (Fig. 5B, C, respectively). A cutoff value of 0.008μm^2^ marks the intersection point of labeled and unlabeled DCV size distributions (Fig. 5D). Only 5% of stained DCVs fall at or below this cutoff value. Thus, vesicles that are smaller than this cutoff are categorized as OXT− in our subsequent analysis.

In EM preparations of regions that contain many OXT+ fibers but few *Oxtr* transcripts, such as the LH and RCH (Fig. 6A, B), OXT+ axons are uniformly non-myelinated and vary in diameter. To determine if oxytocin is likely released from these axons or if they are primarily axons of passage, we examined the LH and RCH using electron microscopy and quantified the incidences of OXT+ axons that contained OXT DCVs. Only 6% of axonal profiles in the LH (n = 48) and 9% of the axonal profiles in the RCH (n = 44) displayed an OXT DCV. When present, DCVs were sparse, indicating that the labeled fibers are more likely axons of passage en route to median eminence (Fig. 6A, B). In contrast, in the MPOA and AHN, many OXT+ profiles contain at least one large DCV (26% of profiles, n = 54 and 23%, n = 39, respectively) (Fig. 6 C, D), suggesting that oxytocin may be released from axons in MPOA and ANH, providing the neuropeptide ligand for the OXTR that is expressed in these regions.

**Fig. 7:**
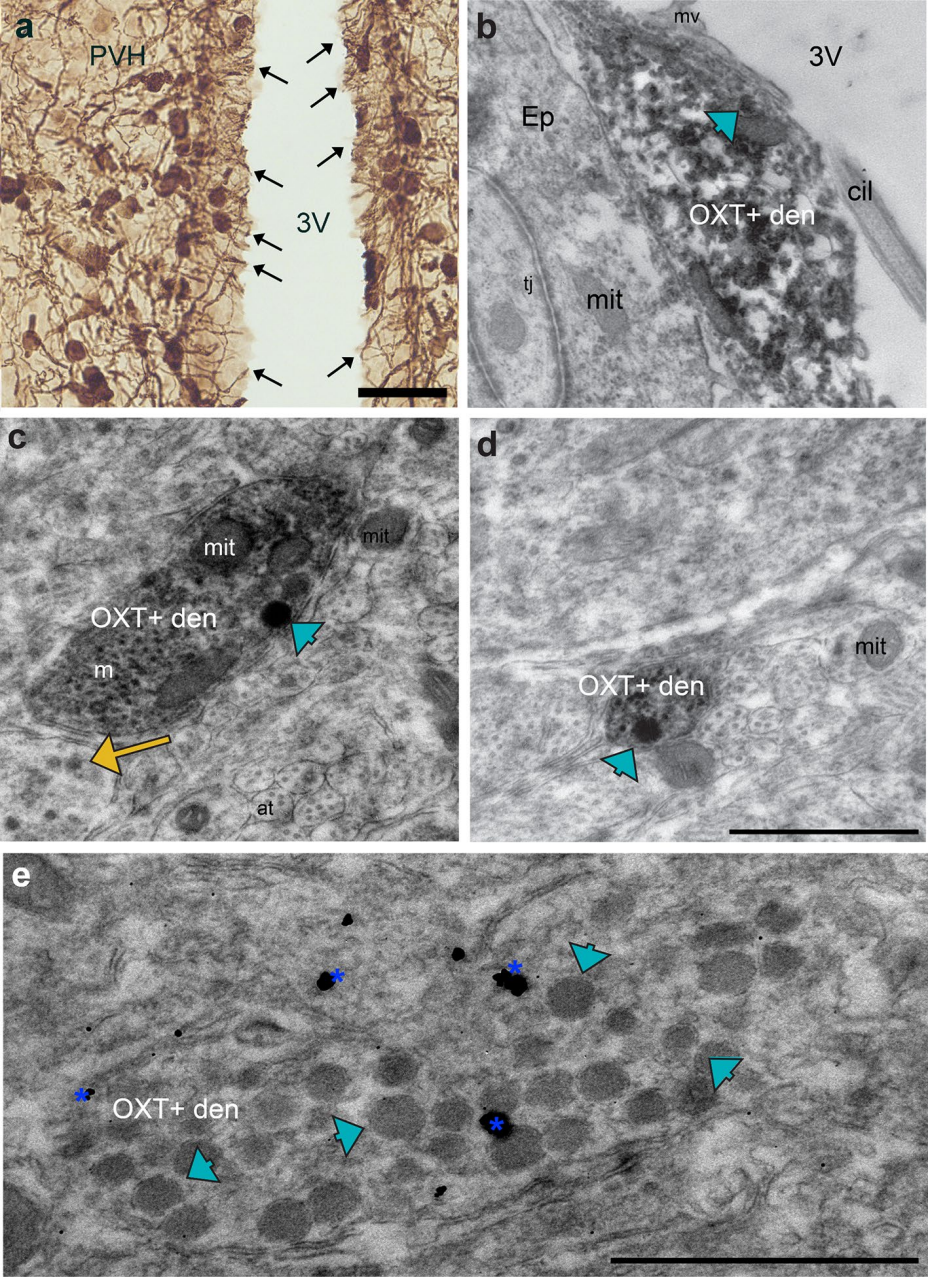
Dendrites of the PVH and SON contain DCVs and are positioned to release OXT into the CSF. **A** OXT+ cells of the PVH extend their dendrites towards the third ventricle (3V), appearing to cross the ependymal cell layer. Scale bar = 500 μm. **B** Electron micrograph of an OXT+ dendrite (OXT+ den) with a DCV (blue arrowhead) located at ventricle-side of the ependymal cells (Ep). The dendrite directly contacts the third ventricle (3 V). **C-D** Dendrites (OXT+ den) within the PVH that contain DCVs (blue arrowhead). Other, smaller DCVs (yellow arrows) are often encountered in unlabeled neuropil in the same region as the OXT+ dendrites and axons. **E** An immuno-gold labeled (blue asterisks) OXT+ dendrite within the SON, with a high density of large DCVs (blue arrowheads). Pixel resolution = 1134.92 pixels/μm; scale bars on B-E = 1 μm. Tj: tight junction of ependymal cell; mit: mitochondria; cil: cilia; mv: microvilli; at: axon terminal; v: vesicles

Regions that contain *Oxtr* transcripts and no OXT+ fibers are especially puzzling, because the source of the oxytocin that could activate OXTR in these regions is not obvious. A possibility is the delivery of oxytocin to cortical extracellular space via CSF circulation. Here, we provide evidence that the circulating CSF may contain OXT that is directly released from dendrites. Using light and electron microscopy, we observed many instances of PVH cell dendrites extending through the ependymal cell layer and directly contacting the third ventricle (Fig. 7A, B). Of the PVH cell dendritic profiles that are in the ependymal zones, 13% (n = 156) contained DCVs (Fig. 7C, D). Thus, these dendrites are situated to exocytose oxytocin from dense-cored vesicles directly into the CSF. Oxytocin may then readily flow through the subarachnoid space along blood capillaries and reach OXTR in the cortical regions via volume transmission.

Finally, using DAB-labelled and gold-enhanced EM we revealed that 40% (N = 30) of dendrites in the SON contain DCVs (Fig. 7E). These dendrites often appear densely packed with large DCVs (Fig. 7E). Interestingly, we noticed using light microscopy, dendrites of the SON cells extend towards the ventral surface of the brain and appear to project through the ependymal cell layer, indicating the possibility that oxytocin can be released into the CSF from SON cells as well (Fig. 2C).

### Whole-Brain imaging of OXT+ staining reveals the extent of ventricular axonal staining

**Fig. 8:**
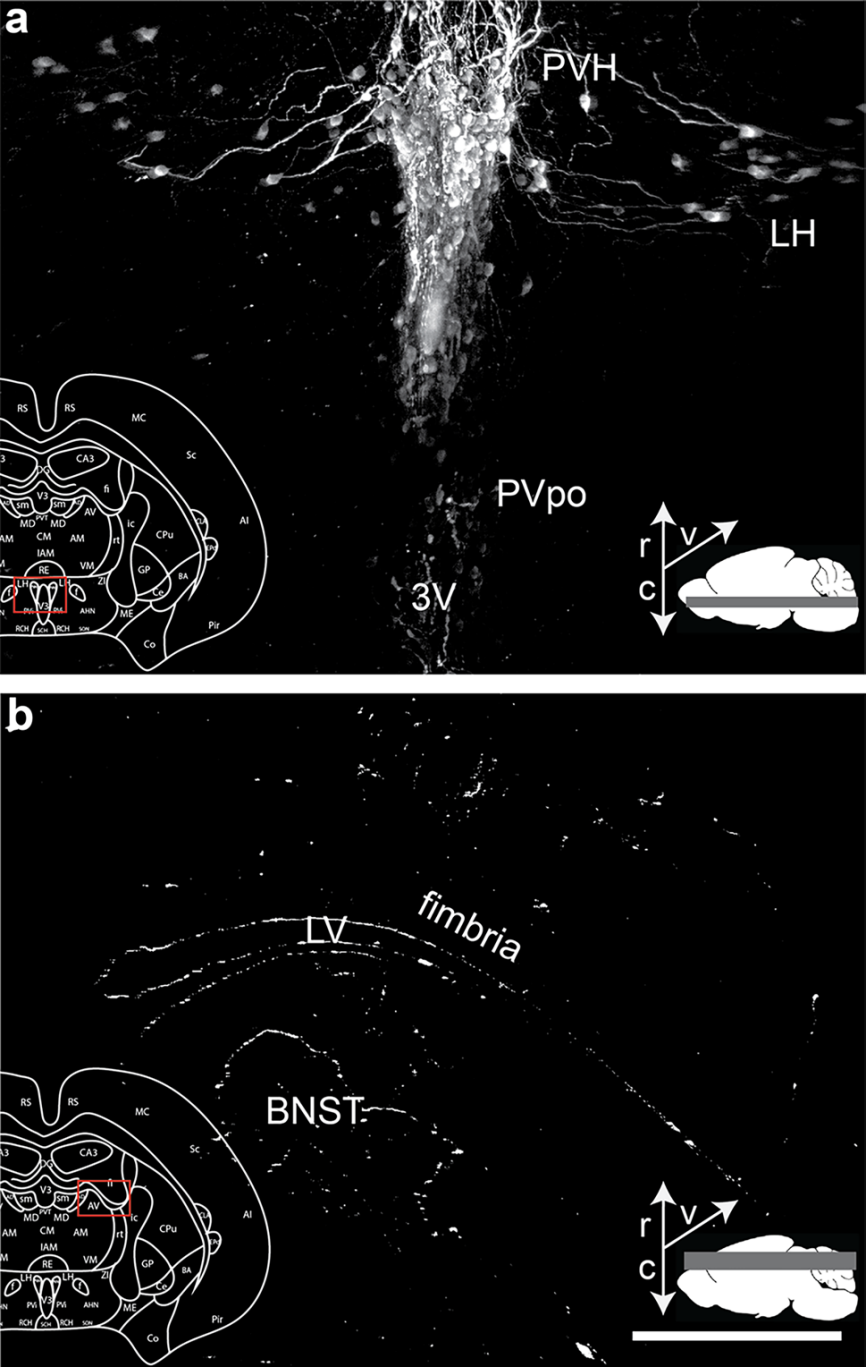
3D whole-brain light-sheet microscopy verified that OXT+ axons border major ventricles. **A** The PVH and PVpo both consistently contain cell bodies, and axons projecting from these regions to follow along the third ventricle (3V), with maximum intensity projection across 775 μm. **B** Representative image of the lateral ventricle (LV), fimbria, and BNST with axonal fibers again bordering a major ventricle, maximum intensity projection across 1554 μm. Both images were derived from 3D whole-brain scan using a light sheet microscope at 5 × objective, are shown in the horizontal plane, and further processed using Imaris software. The superimposed inset marks the region on a corresponding coronal atlas section in red, and thin white arrows in the lower right corner arrows indicate rostral (r), caudal (c), and ventral (v) orientations of the image. Scale bar = 300 μm

To confirm that ventricular OXT labeling is a prominent feature of the prairie vole brain, which was observed via the brightfield microscopy analysis in regions previously mentioned, such as the BNST, and PAG, we examined two brains that were prepared for whole brain clearing and light-sheet microscopy. Major ventricles have positive OXT staining and OXT+ axons appear to follow along ventricles to terminate in subcortical regions. The brains scanned at 5X magnification revealed a strong fluorescence signal lining 3rd ventricle flanked by the hypothalamus (Fig. 8A), as well as the lateral ventricles throughout their anterio-posterior span (Fig. 8B). However, whether these axons are filled with DCVs was not examined using electron microscopy.

## Discussion

The current study provides the first comprehensive mapping of the oxytocinergic system of the prairie vole brain, revealing: 1) The localization of oxytocinergic cell bodies in the prairie vole brain fits the pattern demonstrated in other rodents with a few exceptions, including a prominent OXT+ cell group in the AVPV. 2) The prevalence of oxytocin-carrying axons are correlated with the amount of oxytocin receptor transcripts only in certain subcortical structures including anterior midline thalamic regions, suggesting that oxytocin action in these regions is primarily regulated by neuronal activity and axonal release. 3) While the dorso-lateral stream of oxytocinergic fibers courses through the hypothalamus, these contain only a few dense-cored vesicles, thus are likely axons of passage. The ventro-medial stream, however, contain many DCVs and likely release OXT to act on OXTR in these regions. 4) Similar to other rodent species, the prairie vole cerebral cortex areas are distinct in that they have an abundance of receptors yet no oxytocin axons, suggesting that these regions may utilize oxytocin from sources other than direct axonal release. 5) Finally, both the hypothalamic cell dendrites that protrude through the ependymal cell layer, and OXT+ axons that course along all ventricular surfaces are situated to release oxytocin into the CSF. We posit that CSF is oxytocin’s primary route of access to the receptor-rich cerebral cortex, and potentially other regions of the striatum and pallidum, where the oxytocinergic axons are sparse.

### Comparison of prairie vole to other rodents

Oxytocin-producing cells in the prairie vole brain are in the hypothalamus and the BNST, and this is similar to what has been demonstrated in the mouse (Otero-García et al. 2016; Son et al. 2022). An exception to the general agreement in cell localization among prairie voles and other rodents is one area with OXT cells in the vole: the AVPV. The AVPV is the brain region that is responsible for regulating the estrous cycle and initiating the onset of puberty (Hu et al. 2015; Marraudino et al. 2017). Unlike mice, prairie voles do not display an estrous cycle unless they are induced by exposure to male pheromones (Carter et al. 1987). Thus, the presence of OXT cells in this region may underlie the anatomical basis for the oxytocinergic modulation of this unique function. In addition, prolonged exposure to conspecific pheromones induces oxytocin-dependent pair-bonding behavior in both male and female prairie voles (Williams et al. 1992; Cho et al. 1999; Castro et al. 2022). Whether or not the presence of the oxytocinergic cells in AVPV implies a role for oxytocin in induction of pair-bonding via induced estrous requires further research.

### Humoral release of oxytocin

Similar to other rodents (Brownstein et al. 1980; Brown et al. 2020; Zhang et al. 2021), the axons originating primarily from the prairie vole PVH and SON project to the posterior pituitary. In fact, the axons from the midline PVH take rather a circuitous route to form a girth around the lateral hypothalamus before they join axons from SON and extend ventrally to the median eminence in the midline again. Furthermore, PVH axons display varicosities, intimating that oxytocin may be released from PVH axons (Puder and Papka 2001; Veening et al. 2010; Oti et al. 2021). However, our electron microscopy results are not consistent with the possibility that oxytocin is released from dense cored vesicles of PVH axons en route to the posterior pituitary. These varicosities may be a characteristic structural feature of OXT cells and axons, as has long been observed in OXT immunostained tissue (Theodosis 1985; Morris and Pow 1988; Veening et al. 2010), but may not necessarily indicate release.

### Axonal release of oxytocin on select brain regions

Other regions of the hypothalamus with many fibers and an abundance of axonal swellings such as the AHN and MPOA contain dense cored vesicles, situated to release oxytocin, presumably via the neuronal activity that occurs at their cell bodies. Many other regions, including the AcbSh, RE of the thalamus, and olfactory cortex display oxytocin axons with similar morphological features and as such may also contain DCVs to release OXT directly. This is consistent with previous studies indicating that the release of oxytocin can be activity-dependent and will act quickly to modulate the activity of regions that contain oxytocin fibers and receptors (Ludwig et al. 2002; Rossoni et al. 2008; Knobloch et al. 2012; Chini et al. 2017; Carcea et al. 2021). The direct release of OXT in these areas can bind to OXTR to impact unique prairie vole behaviors such as social monogamy and affiliative interactions (Ross et al. 2009; Ophir et al. 2012). The origins of oxytocinergic cells that provide these axons, and how those cells are activated to release their peptide warrant further investigation (Zhang et al. 2021; Li et al. 2024). For example, a recent study has demonstrated, in the mouse, that PVH cells that innervate subcortical limbic regions such as the amygdala and hindbrain regions are a subpopulation distinct from those projecting to the pituitary (Li et al. 2024), suggesting that activity-dependent release of oxytocin in central regions may be independent of the conditions that lead to humoral release of oxytocin.

It was suggested that the low incidence of oxytocin fibers in the cerebral cortex may be a state-dependent observation, and that the oxytocin production surge in the hypothalamus during the birthing process may render cortical axons that otherwise contain low amounts of the neuropeptide detectable. In support of this idea, work in the mouse model has shown the presence of OXT+ fibers, albeit sparsely, in the auditory cortex of dams (Marlin et al. 2015), and suggested that axonally released oxytocin in this region may regulate maternal behavior that relies on sensory discrimination of pup calls. Future studies should examine in the prairie vole if, under certain circumstances, OXT fibers can be observed in other cortical regions as well.

### Oxytocin in the CSF

Oxytocin’s presence in the CSF has been acknowledged in many studies (Mens et al. 1983; Veening et al. 2010; Kagerbauer et al. 2013; Jurek and Neumann 2018). Furthermore, the CSF is confluent with the extracellular fluid of the neuropil through intercellular junctions between ependymal cells, it has been suggested that oxytocin might reach its receptor via volume transmission from the CSF (Ludwig et al. 2002; Veening et al. 2010). However, how oxytocin gets to the CSF has been the subject of intense debate. One source of the CSF oxytocin could be the blood. Humorally released oxytocin may return to cerebral blood circulation and diffuse into the CSF (Lee et al. 2018; Yamamoto et al. 2019). However, one study found only about 0.002% of peripherally applied OXT reaches the CNS following IV injections (Mens et al. 1983), and later work has found similar results, (Freeman et al. 2016; Lee et al. 2018), suggesting that blood is not the primary source of the CSF oxytocin (although, see Yamamoto et al. 2019). Other evidence also supports the idea that CSF oxytocin may have a primary origin other than the blood (Ludwig et al. 2002; Leng and Ludwig 2008; Veening et al. 2010; Kagerbauer et al. 2013; Lefevre et al. 2017). First, oxytocin concentration in the CSF of humans is consistently higher than in plasma and increases in plasma oxytocin is not a predictor of increases in the CSF oxytocin (Kagerbauer et al. 2013). In addition, the plasma oxytocin has a significantly shorter half-life (about 2–5 min) than the CSF oxytocin (about 20–30 min) (Mens et al. 1983; Leng and Ludwig 2008; Veening et al. 2010), suggesting that plasma clearance rate may pose a bottleneck for sustaining the CSF oxytocin concentration.

Then, how does endogenous, central oxytocin reach the CSF? Our results provide further evidence that the dendrites of the oxytocinergic cells in the hypothalamus are ideally positioned to release oxytocin directly into the CSF. We have demonstrated that OXT+ dendrites frequently cross the ependymal cell layer and directly contact the ventricle. The medial hypothalamic cells that project to the posterior pituitary are situated in an opportune position because their dendrites readily line the ventricular surface and, in many instances, protrude into the ventricles, potentially providing a basal amount of oxytocin in the CSF.

The conditions that are associated with large hormonal surges, such as birth, within the periphery also likely influence the oxytocin concentration in the CSF. For example, during labor, there is a pulsatile release of OXT into the periphery in amounts much higher than seen pre-pregnancy (Leake et al. 1981). Similarly, the peripheral release of oxytocin during gestation is high (Uvnäs-Moberg et al. 2019), and this may increase the central release of oxytocin into the CSF via dendritic exocytosis. This is particularly important because birth triggers many oxytocin-mediated central behaviors including the onset of maternal behavior in rodents (Numan 1988; Stolzenberg and Champagne 2016) and plasticity within cortical regions (Marlin et al. 2015; Mitre et al. 2016). Further, birth and exogenous oxytocin treatment trigger epigenetic changes to the oxytocin receptor that primes the system to prepare the animal for motherhood (Stolzenberg and Champagne 2016; Danoff et al. 2023). Thus, the birth-triggered robust plasticity within cortical regions must be mediated via oxytocin circulating within the CSF, and the release of oxytocin from PVH cell dendrites along the ependymal cell layer is the most likely source of the CSF oxytocin.

Ependymal cells not only contribute to CSF production and movement, but they also sift through the circulating fluid to clear accumulated waste and to maintain homeostasis between the extracellular space and CSF (Deng et al. 2023). Ependymal cells have also been found to contain transporters, such as the glucose transporter indicating they may play a critical role in the transport of essential substances between the extracellular space and the ventricles (Murakami et al. 2016; Deng et al. 2023). Therefore, the ependymal cells could play an active role in transporting oxytocin exocytosed from oxytocinergic dendrites from the extracellular space into the CSF. It should also be noted that oxytocin release into CSF can happen not only at the 3rd ventricle surrounded by the hypothalamus but also along the entire ventricular surface, due to an extensive stream of oxytocinergic axons as well as ectopic oxytocinergic cells that are located by the ventricles, something that has also been observed in the mouse model recently (Son et al. 2022). The presence of DCVs in the axons that border all major ventricles should be determined in future studies. Whether or not the dendritic and axonal oxytocin release into CSF is triggered by similar central activity also requires further examination.

### Are there multiple oxytocinergic systems?

Our results highlight three different release modes of oxytocin: humoral release to the pituitary, axonal release, mostly in subcortical structures, and the dendritic, and potentially axonal release into ventricles. These three modes of release may differentially contribute to the diversity of oxytocin’s action in the periphery and the central nervous system. For example, oxytocin’s physiological effects on the body such as the initiation of contractions at birth and milk letdown during lactation (Soloff et al. 1979; Perkinson et al. 2021) is purely a function of oxytocin that is synthesized primarily in the magnocellular cells of PVH and SON (Vigneaud et al. 1953; Brownstein et al. 1980; Zhang et al. 2021; Li et al. 2024) and released in the pituitary, although there is also evidence for local synthesis of oxytocin in peripheral tissue as well (Einspanier and Ivell 1997; Jankowski et al. 1998; Carter et al. 2020). On the other hand, modifying the behaviors related to bonding and the stress response involves axonal oxytocin release in limbic regions, including the nucleus accumbens, the amygdala, and bed nucleus of the stria terminalis. The oxytocin-releasing axons in these regions originate from the PVH, (Zhang et al. 2021; Freda et al. 2022; Li et al. 2024) and, as we also demonstrated in the current study, they release oxytocin from axonal varicosities (Armstrong et al. 1982; Tweedle et al. 1989; Knobloch et al. 2012) to bind OXTR.

Finally, oxytocin’s role in regulating aggressive behaviors, learning, and other more complex cognitive functions that require the involvement of sensory and association cortices (Bales and Carter 2003; Hurlemann et al. 2010; Marlin et al. 2015; Jurek and Neumann 2018). For example, maternal behavior in mice, which is typically displayed only after the oxytocin surge associated with giving birth, relies on plasticity within cortical regions and oxytocin to OXTR binding (Marlin et al. 2015; Froemke and Young 2021; Carcea et al. 2021). Similarly, the prairie vole displays consoling behavior after partner stress and separation which is also linked to OXTR binding in the prelimbic cortex (PrL) and anterior cingulate cortex (Cg), accentuating the importance of the availability of oxytocin to the cortex (Burkett et al. 2016). However, while many cortical regions contain an abundance of OXTR (Bosch et al. 2016; Newmaster et al. 2020; Inoue et al. 2022; Son et al. 2022) they lack a high density of oxytocin-containing fibers (Mitre et al. 2018; Liao et al. 2020; Manjila et al. 2022; Son et al. 2022), as demonstrated in the current study for the prairie voles, suggesting that oxytocin’s effect in the cortex is independent of axonal or humoral oxytocin release (Veening et al. 2010; Ferris et al. 2015; Mitre et al. 2016; Son et al. 2022). The results of the current study demonstrate structural evidence for substantial oxytocin release into the CSF and suggest that this third mode of oxytocin release may be the main source of oxytocin that mediates aggression, learning and other higher cognitive functions in the cerebral cortex, where the axonal release of oxytocin is unpronounced.

## Supplementary Information

Below is the link to the electronic supplementary material.

Supplementary file1 Illustration of the creation of the vole brain atlas. A) A myelin-stained section at oc +1100 μm with areas that were able to be demarcated by using the myelin section outlined and labeled, black boxed region is magnified to 20x in D. B) The Nissl section 100 μm anterior to the myelin stain with the areas that were outlined in the myelin stain and regions that could be further outlined using the Nissl stain, such as the SON, again magnified region shown in E. C) The OXT-stained section that lies between the myelin and Nissl stain with the combined outlines overlaid, magnified region shown in F. A-C) Scale bar = 500 μm, D-F) scale bar = 50 μm (PNG 13818 KB)

Supplementary file2 Few sexual dimorphisms in OXT+ cell bodies and axons are seen between male and female prairie voles. In both plots, the x-axis illustrates the t-statistic where positive values indicate higher mean scores in females and negative values indicate higher mean scores in males. The y-axis represents the negative logarithm (base 10) of the p-values with higher values indicating greater statistical significance. Brain regions with p-values < 0.05 are indicated with a red circle and highlight significant differences. All non-significant regions are displayed with gray circles. A) There is not a significant difference in OXT+ cell body density in males vs. Females in any brain regions examined (examined using a Welch’s t-test). B) Although there is not sufficient statistical power, two regions, the EPd (p = 0.007) and PrL (p = 0.02), may be sexually dimorphic, with males (EPd: Mean = 0.17 ± SD = 0.026, PrL: 0.25±0.07) showing higher SDI values than females (EPd: 0 ± 0, PrL: 0.04 ± 0.06) (PNG 332 KB)

Supplementary file3 (DOCX 17 KB)

Supplementary file4 (DOCX 34 KB)

Supplementary file5 (DOCX 27 KB)

## Data Availability

All raw data for SDI values for cell bodies and axons for each animal are provided as Supplemental Tables 1–2 with this manuscript. Supplemental Table 3 provides pooled SDI vs. Oxtr for regions both included and excluded from the analysis. The archive of anatomical images is available for viewing upon request.

## Abbreviations

AcbC: Nucleus accumbens core
AcbSh: Nucleus accumbens shell
acoc: Anterior commissure, olfactory limb
act: Anterior commissure, temporal limb
AD: Anterodorsal nucleus of the thalamus
AHN: Anterior hypothalamic nucleus
AI: Agranular insular area
AM: Anteromedial nucleus of the thalamus
Amy: Amygdala
aq: Cerebral aqueduct
AV: Anteroventral nucleus of the thalamus
AVPV: Anteroventral periventricular nucleus
BA: Basomdeial/basolateral amygdalar nucleus
BNST: Bed nucleus of the stria terminalis
CA1: Hippocampal area 1
CA2: Hippocampal area 2
CA3: Hippocampal area 3
cc: Corpus callosum
Ce: Central amygdalar nucleus
cg: Cingulum bundle
Cg: Cingulate cortex
CLA: Claustrum
CM: Central medial thalamic nucleus
Co: Cortical amygdaloid nucleus
CPu: Caudate putamen
DB: Diagonal band of Broca
DG: Dentate gyrus
DR: Dorsal raphe
EPd: Endopiriform dorsal part
f: Fornix
GL: Glomerular layer of the olfactory bulb
GP: Globus pallidus
GrO: Granular cell layer olfactory limb
islm: Major island of Calleja
LA: Lateral amygdalar nucleus
LPOA: Lateral preoptic area
LS: Lateral septum
LSd: Lateral septum-dorsal
LSi: Lateral septum-intermediate
LSv: Lateral septum-ventral
LV: Lateral ventricle
MC: Motor cortex
ME: Median eminence
Me: Medial amygdalar nucleus
MEPO: Median preoptic nucleus
MM: Mammillary nucleus
MPOA: Medial preoptic area
MS: Medial septal nucleus
och: Optic chiasm
opt: Optic tract
ORB: Orbital area
OT: Olfactory tubercle
PAG: Periaqueductal gray
PH: Posterior hypothalamic nucleus
Pir: Piriform cortex
Pons: Pons
PrL: Prelimbic cortex
PVH: Paraventricular hypothalamic nucleus
PVi: Periventricular hypothalamic nucleus-intermediate
PVpo: Periventricular hypothalamic preoptic part
PVT: Paraventricular nucleus of the thalamus
RCH: Retrochiasmatic area
RE: Nucleus of reuniens
RH: Rhomboid nucleus
RS: Retrosplineal area
rt: Reticular nucleus of the thalamus
RTm: Reticular nucleus -midbrain
Sc: Somatosensory cortex
SC: Superior colliculus
SCH: Suprachiasmatic nucleus
sm: Stria medularis
SN: Substantia nigra
SNR: Substantia nigra reticular part
SON: Supraoptic nucleus
st: Stria terminalis
SUB: Subiculum
TT: Taenia tecta
Tu: Tuberal nucleus
V3: Third ventricle
VLPO: Ventrolateral preoptic area
VM: Ventral medial thalamic nucleus
VMH: Ventromeidal hypothalamus
VMPO: Ventromedial preoptic area
VP: Ventral pallidum
VTA: Ventral tegmental area
ZI: Zona incerta
