# Supplementary figures and images for: The central oxytocinergic system of the prairie vole
Source: Brain Struct Funct. 2024 Jul 23;229(7):1737–56. doi: 10.1007/s00429-024-02832-1 (PMC11374920; doi:10.1007/s00429-024-02832-1)

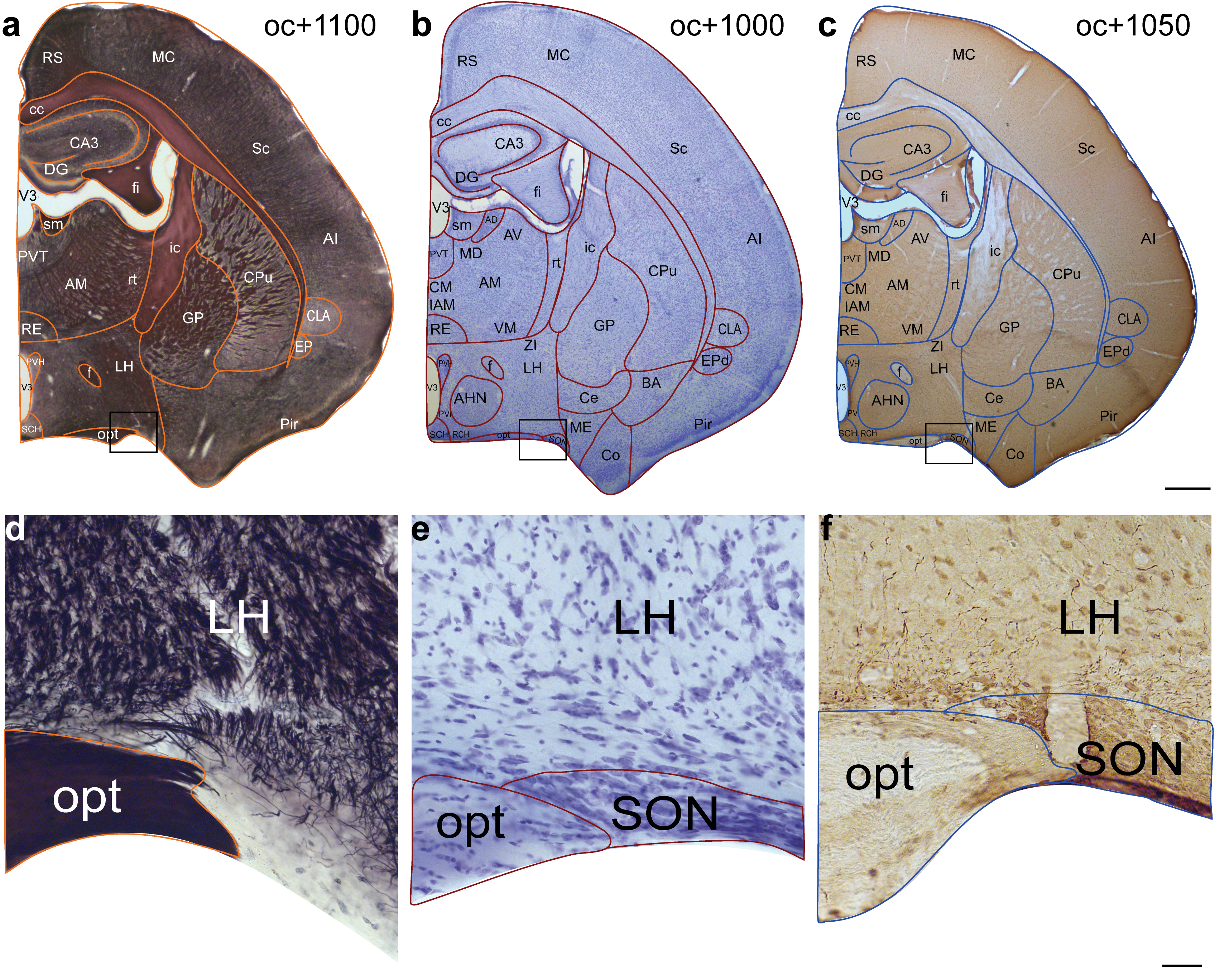

Supplement: Supplementary file 1 — Supplementary file1 Illustration of the creation of the vole brain atlas. A) A myelin-stained section at oc +1100 μm with areas that were able to be demarcated by using the myelin section outlined and labeled, black boxed region is magnified to 20x in D. B) The Nissl section 100 μm anterior to the myelin stain with the areas that were outlined in the myelin stain and regions that could be further outlined using the Nissl stain, such as the SON, again magnified region shown in E. C) The OXT-stained section that lies between the myelin and Nissl stain with the combined outlines overlaid, magnified region shown in F. A-C) Scale bar = 500 μm, D-F) scale bar = 50 μm (PNG 13818 KB) [file 429_2024_2832_MOESM1_ESM.png]

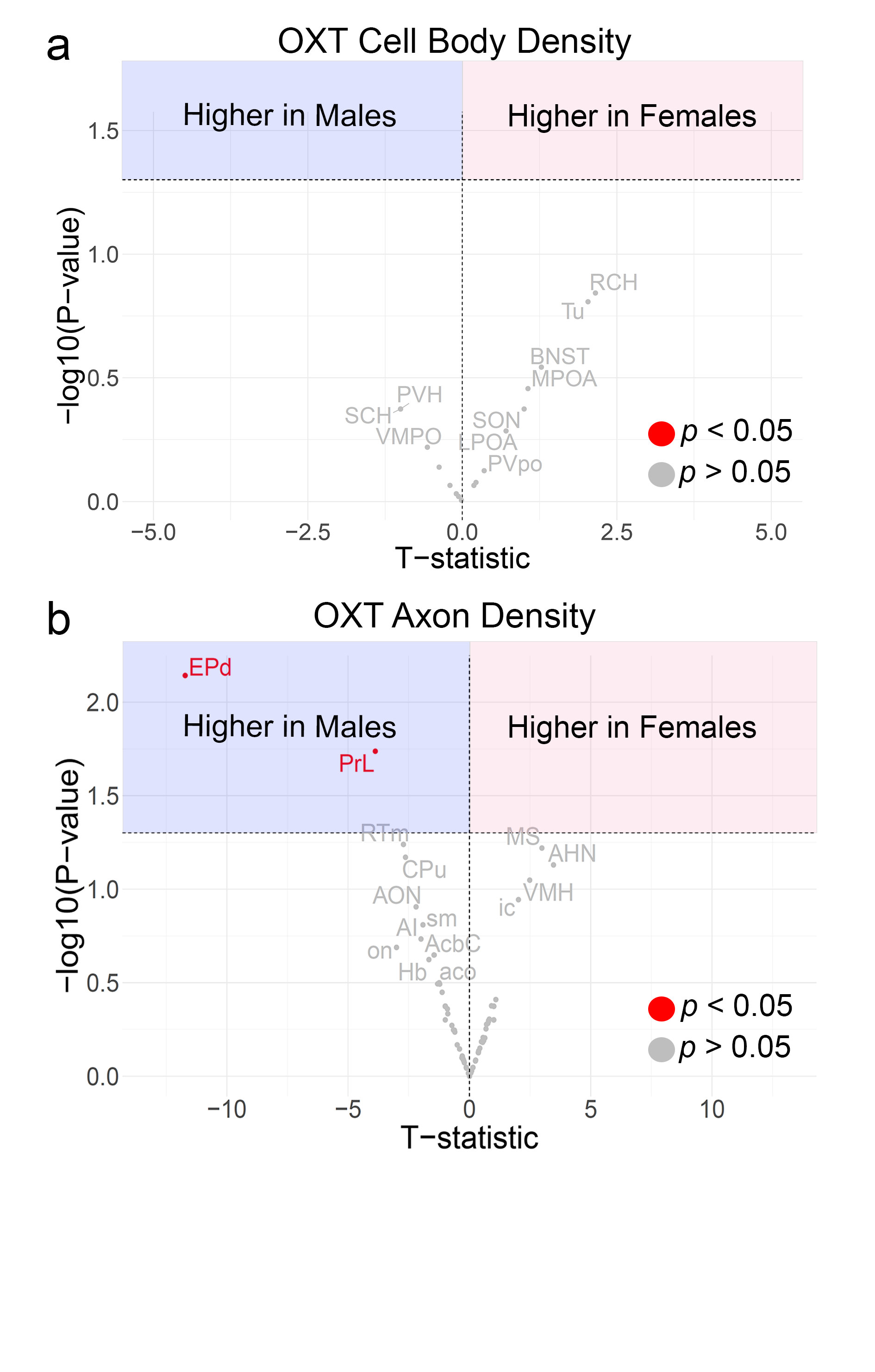

Supplement: Supplementary file 2 — Supplementary file2 Few sexual dimorphisms in OXT+ cell bodies and axons are seen between male and female prairie voles. In both plots, the x-axis illustrates the t-statistic where positive values indicate higher mean scores in females and negative values indicate higher mean scores in males. The y-axis represents the negative logarithm (base 10) of the p-values with higher values indicating greater statistical significance. Brain regions with p-values < 0.05 are indicated with a red circle and highlight significant differences. All non-significant regions are displayed with gray circles. A) There is not a significant difference in OXT+ cell body density in males vs. Females in any brain regions examined (examined using a Welch’s t-test). B) Although there is not sufficient statistical power, two regions, the EPd (p = 0.007) and PrL (p = 0.02), may be sexually dimorphic, with males (EPd: Mean = 0.17 ± SD = 0.026, PrL: 0.25±0.07) showing higher SDI values than females (EPd: 0 ± 0, PrL: 0.04 ± 0.06) (PNG 332 KB) [file 429_2024_2832_MOESM2_ESM.png]
