# Supplementary material for: The central oxytocinergic system of the prairie vole
Source: Brain Struct Funct. 2024 Jul 23;229(7):1737–56. doi: 10.1007/s00429-024-02832-1 (PMC11374920; doi:10.1007/s00429-024-02832-1)
Supplement: Supplementary file 3 — Supplementary file3 (DOCX 17 KB) [file 429_2024_2832_MOESM3_ESM.docx]

**Supplemental Table 1**

Staining density index scores for all OXT cell-body containing regions of each male and female subject. SDI was calculated as: (3N3+2N2+N1)/3NT, where N3, N2, and N1 are the number of sections with a +++, ++, or + score, and NT is the number of sections the region spans, including the sections with no cells. The values in this table were used for the analysis in Supplemental Figure 2(a). NA's indicate the region was not examined in the subsample of sections in that animal.

| **Abbreviation** | **Full Region Name** | **Male 1 SDI** | **Male 2 SDI** | **Male 3 SDI** | **Female 1 SDI** | **Female 2 SDI** | **Female 3 SDI** |
| --- | --- | --- | --- | --- | --- | --- | --- |
| AHN | anterior hypothalamic nucleus | 0.22 | 0.00 | 0.17 | 0.22 | 0.00 | 0.22 |
| ARC | arcuate nucleus | 0.17 | 0.00 | 0.00 | NA | NA | 0.00 |
| AVPV | anteroventral periventricular nucleus | 0.22 | 1.00 | 0.67 | 0.33 | 1.00 | 0.17 |
| BNST | bed nucleus of the stria terminalis | 0.28 | 0.33 | 0.00 | 0.33 | 0.83 | 0.25 |
| DM | dorsal medial nucleus of the hypothalamus | 0.08 | 0.33 | NA | NA | 0.00 | 0.33 |
| LH | lateral hypothalamus | 0.50 | 0.53 | 0.22 | 0.44 | 0.56 | 0.33 |
| LPOA | lateral preoptic area | 0.17 | 0.00 | 0.00 | 0.00 | 0.17 | 0.17 |
| MEPO | median preoptic area | 0.17 | 0.33 | 0.33 | 0.33 | 0.33 | 0.17 |
| MPOA | medial preoptic area | 0.44 | 0.33 | 0.00 | 0.67 | 0.33 | 0.33 |
| PVH | paraventricular hypothalamic nucleus | 1.00 | 1.00 | 1.00 | 0.89 | 1.00 | 1.00 |
| PVi | periventricular hypothalamic nucleus-intermediate | 0.11 | 0.17 | 0.17 | 0.33 | 0.00 | 0.08 |
| PVpo | periventricular hypothalamic nucleus- preoptic part | 0.67 | 0.33 | 0.00 | 0.33 | 0.33 | 0.56 |
| RCH | retrochiasmatic nucleus | 0.29 | 0.17 | NA | 0.67 | 1.00 | 0.33 |
| SCH | suprachiasmatic nucleus | 0.11 | 0.00 | 0.00 | 0.00 | 0.00 | 0.00 |
| SON | supraoptic nucleus | 0.97 | 1.00 | 1.00 | 1.00 | 1.00 | 1.00 |
| Tu | (tuberal nucleus) | 0.56 | 0.67 | 0.50 | NA | 0.67 | 0.78 |
| VLPO | ventrolateral preoptic area | 0.06 | 0.67 | 0.00 | 0.00 | 0.67 | 0.00 |
| VMPO | ventromedial preoptic area | 0.20 | 1.00 | 0.33 | 0.00 | 0.33 | 0.67 |
