# Supplementary material for: The central oxytocinergic system of the prairie vole
Source: Brain Struct Funct. 2024 Jul 23;229(7):1737–56. doi: 10.1007/s00429-024-02832-1 (PMC11374920; doi:10.1007/s00429-024-02832-1)
Supplement: Supplementary file 4 — Supplementary file4 (DOCX 34 KB) [file 429_2024_2832_MOESM4_ESM.docx]

**Supplemental Table 2**

Staining Density Index scores for OXT axonal density in regions of each male and female subject. SDI was calculated as: (3N3+2N2+N1)/3NT, where N3, N2, and N1 are the number of sections with a +++, ++, or + score, and NT is the number of sections the region spans, including the sections with no cells. The values in this table were used for the analysis in Supplemental Figure 2(b). NA's indicate the region was not examined in the subsample of sections in that animal.

| **Abbreviation** | **Full Region Name** | **Male 1 SDI** | **Male 2 SDI** | **Male 3 SDI** | **Female 1 SDI** | **Female 2 SDI** | **Female 3 SDI** |
| --- | --- | --- | --- | --- | --- | --- | --- |
| ac | anterior commissure | 0.33 | 0.44 | 0.17 | 0.00 | 0.67 | 0.33 |
| AcbC | nucleus accumbens core | 0.48 | 0.78 | 1.00 | 0.00 | 0.50 | 0.67 |
| AcbSh | nucleus accumbens shell | 0.54 | 0.89 | 1.00 | 0.33 | 1.00 | 0.67 |
| aco | anterior commissure, olfactory limb | 0.19 | 0.08 | 0.00 | 0.00 | 0.00 | 0.06 |
| act | anterior commissure, temporal limb | 0.22 | NA | NA | NA | NA | NA |
| AD | anterodorsal nucleus of the thalamus | 0.00 | 0.00 | 0.00 | 0.00 | 0.00 | 0.00 |
| AHN | anterior hypothalamic nucleus | 0.78 | 0.89 | 0.67 | 1.00 | 1.00 | 1.00 |
| AI | agranular insular area | 0.06 | 0.00 | 0.07 | 0.00 | 0.00 | 0.00 |
| AM | anteromedial nucleus of the thalamus | 0.07 | 0.00 | 0.00 | 0.00 | 0.33 | 0.00 |
| Amy | amygdala | 0.23 | 0.50 | 0.44 | 0.22 | 0.78 | 0.38 |
| AOB | accessory olfactory bulb | 0.00 | NA | NA | NA | NA | NA |
| AON | anterior olfactory nucleus | 0.27 | 0.89 | 0.67 | 0.11 | 0.33 | 0.08 |
| aq | cerebral aqueduct | 0.67 | 0.00 | 0.00 | 0.00 | 0.00 | NA |
| ARC | arcuate nucleus | 0.67 | 1.00 | 1.00 | NA | NA | 1.00 |
| Au | auditory cortex | 0.02 | 0.00 | 0.11 | NA | 0.00 | 0.07 |
| AV | anteroventral nucleus of the thalamus | 0.11 | 0.00 | 0.00 | 0.00 | 0.00 | 0.00 |
| AVPV | anteroventral periventricular nucleus | 0.72 | 1.00 | 1.00 | 0.67 | 1.00 | 1.00 |
| BNST | bed nucleus of the stria terminalis | 0.61 | 1.00 | 1.00 | 0.67 | 1.00 | 0.83 |
| bsc | brachium of superior colliculus | 0.00 | 0.00 | 0.00 | 0.00 | 0.00 | 0.00 |
| CA1 | hippocampal area 1 | 0.02 | 0.00 | 0.00 | 0.00 | 0.00 | 0.00 |
| CA2 | hippocampal area 2 | 0.00 | 0.00 | 0.00 | NA | 0.00 | 0.00 |
| CA3 | hippocampal area 3 | 0.10 | 0.00 | 0.00 | 0.00 | 0.00 | 0.00 |
| cc | corpus callosum | 0.04 | 0.00 | 0.00 | NA | 0.00 | 0.03 |
| cg | cingulum bundle | 0.00 | 0.00 | NA | 0.00 | 0.00 | 0.00 |
| Cg | cingulate cortex | 0.02 | 0.08 | 0.00 | 0.00 | 0.00 | 0.00 |
| Cga | cingulate area-anterior | 0.07 | 0.00 | 0.00 | 0.00 | NA | 0.00 |
| CLA | claustrum | 0.08 | 0.00 | 0.00 | 0.00 | 0.00 | 0.00 |
| CM | central medial thalamic nucleus | 0.35 | 0.40 | 0.33 | 0.22 | 1.00 | 0.60 |
| Co | cortical amygdaloid nucleus | 0.08 | 0.00 | NA | NA | 0.00 | 0.00 |
| cpd | cerebral peduncle | 0.13 | 0.33 | 0.00 | 0.00 | 0.50 | 0.22 |
| CPu | caudate putamen | 0.20 | 0.11 | 0.17 | 0.06 | 0.11 | 0.06 |
| csc | superior colliculus commmisure | 0.61 | 0.33 | 0.67 | 0.00 | 1.00 | 0.33 |
| DB | diagonal band of Broca | 0.29 | 0.44 | 0.33 | 0.17 | 1.00 | 0.50 |
| DG | dentate gyrus | 0.02 | 0.00 | 0.00 | 0.00 | 0.00 | 0.00 |
| dhc | dorsal hippocampal commissure | 0.00 | 0.00 | 0.00 | NA | 0.00 | 0.00 |
| DLGN | dorsal lateral geniculate nucleus | 0.00 | 0.00 | 0.00 | NA | NA | 0.00 |
| DM | dorsal medial nucleus of the hypothalamus | 0.75 | 1.00 | NA | NA | 1.00 | 1.00 |
| DR | dorsal raphe | 0.50 | 0.67 | NA | NA | NA | NA |
| ec | external capsule | 0.00 | 0.00 | NA | NA | NA | 0.00 |
| emt | external medullary lamina of the thalamus | 0.00 | 0.00 | 0.00 | NA | 0.00 | 0.00 |
| Ent | entorhinal cortex | 0.00 | 0.00 | 0.00 | 0.00 | 0.00 | 0.00 |
| EPd | endopiriform dorsal part | 0.15 | 0.20 | 0.17 | NA | 0.00 | 0.00 |
| f | fornix | 0.39 | 0.92 | 0.83 | 0.67 | 0.80 | 0.80 |
| fa | corpus callosum- anterior forceps | 0.33 | 0.00 | 0.00 | 0.00 | NA | 0.67 |
| fi | fimbria | 0.04 | 0.00 | 0.00 | 0.00 | 0.00 | 0.17 |
| fr | fasciculus retroflex | 0.13 | 0.67 | 0.00 | NA | 0.67 | 0.11 |
| FrA | frontal association cortex | 0.10 | 0.00 | NA | NA | 0.33 | NA |
| GL | glomerular layer of the olfactory bulb | 0.00 | NA | NA | NA | NA | NA |
| GP | globus pallidus | 0.29 | 0.22 | 0.67 | 0.22 | 0.67 | 0.20 |
| GrO | granular cell layer olfactory limb | 0.17 | NA | NA | 0.00 | 0.17 | 0.00 |
| Hb | habenula | 0.15 | 0.00 | 0.33 | 0.00 | 0.00 | 0.00 |
| IAM | interanteromedial nucleus of the thalamus | 0.00 | NA | NA | NA | NA | NA |
| ic | internal capsule | 0.44 | 0.67 | 0.33 | 0.78 | 0.92 | 0.58 |
| IL | infralimbic cortex | 0.24 | 0.33 | 0.00 | 0.00 | 0.50 | 0.17 |
| IMD | intermediodorsal nucleus of the thalamus | 0.33 | 0.67 | 0.33 | 0.00 | NA | 0.67 |
| IP | interpeduncular nucleus | 0.33 | 1.00 | NA | NA | 1.00 | 1.00 |
| islm | major island of Calleja | 0.33 | NA | NA | NA | NA | NA |
| LD | lateral dorsal thalamic nucleus | 0.00 | 0.00 | 0.00 | 0.00 | 0.00 | 0.00 |
| LGN | lateral geniculate nucleus | 0.00 | 0.00 | NA | NA | 0.00 | 0.00 |
| LH | lateral hypothalamic nucleus | 0.98 | 1.00 | 1.00 | 1.00 | 1.00 | 1.00 |
| LHb | lateral habenular nucleus | 0.17 | NA | NA | NA | NA | NA |
| lot | lateral olfactory tract | 0.00 | 0.00 | 0.00 | 0.00 | 0.00 | 0.00 |
| LP | lateral posterior thalamic nucleus | 0.00 | 0.00 | 0.00 | NA | NA | 0.00 |
| LPOA | lateral preoptic area | 0.61 | 0.67 | 0.67 | 0.00 | 1.00 | 1.00 |
| LS | lateral septum | 0.37 | 0.83 | 0.33 | 0.17 | 0.67 | 0.67 |
| LSd | lateral septum - dorsal | 0.72 | 1.00 | 0.67 | 0.00 | 1.00 | 0.33 |
| LSi | lateral septum - intermediate | 0.67 | NA | NA | NA | Na | 0.67 |
| LSv | lateral septum - ventral | 0.56 | 1.00 | 0.33 | 0.33 | 1.00 | 0.67 |
| LV | lateral ventricle | 0.35 | 0.00 | 0.00 | 0.00 | 0.00 | 0.00 |
| MC | motor cortex | 0.01 | 0.00 | 0.00 | 0.00 | 0.00 | 0.00 |
| MD | mediodorsal nucleus of the thalamus | 0.03 | 0.00 | 0.00 | 0.00 | 0.00 | 0.00 |
| ME | median eminence | 1.00 | 1.00 | NA | NA | NA | 1.00 |
| MEPO | median preoptic nucleus | 0.83 | 1.00 | 1.00 | 1.00 | 1.00 | 0.83 |
| MGN | medial geniculate nucleus | 0.21 | 0.11 | 0.00 | 0.00 | 0.17 | 0.00 |
| MHb | medial habenular nucleus | 0.17 | NA | NA | NA | NA | NA |
| ml | medial lemniscus | 0.04 | 0.00 | 0.00 | NA | 0.00 | 0.00 |
| MM | mammillary nucleus | 0.78 | 1.00 | 1.00 | NA | 1.00 | 0.67 |
| MPOA | medial preoptic area | 0.69 | 1.00 | 1.00 | NA | 1.00 | 1.00 |
| MS | medial septal nucleus | 0.58 | 0.67 | 0.33 | NA | 1.00 | 0.83 |
| mtt | mammillothalamic tract | 0.14 | 0.22 | 0.00 | NA | 0.33 | 0.07 |
| och | optic chiasm | 0.19 | NA | NA | NA | 0.00 | 0.00 |
| on | optic nerve | 0.67 | 0.33 | NA | NA | 0.00 | 0.00 |
| opt | optic tract | 0.35 | 0.28 | 0.44 | 0.22 | 0.44 | 0.33 |
| ORB | orbital area | 0.30 | 0.33 | 0.00 | 0.00 | 0.08 | 0.13 |
| OT | olfactory tubercle | 0.27 | 0.47 | 0.33 | 0.11 | 0.78 | 0.40 |
| PAG | periaqueductal gray | 0.92 | 1.00 | 1.00 | 0.67 | 1.00 | 1.00 |
| PF | parafascicular thalamic nucelus | 0.17 | 0.00 | 0.00 | NA | NA | 0.00 |
| PH | posterior hypothalamic nucleus | 0.67 | 1.00 | 1.00 | NA | 1.00 | 1.00 |
| Pir | piriform cortex | 0.14 | 0.00 | 0.00 | 0.00 | 0.00 | 0.04 |
| Po | posterior complex of the thalamus | 0.00 | 0.00 | 0.00 | 0.00 | 0.00 | 0.00 |
| Pons | Pons | 0.58 | 1.00 | 1.00 | 1.00 | 1.00 | NA |
| PRh | perirhinal cortex | 0.02 | 0.00 | 0.00 | 0.00 | 0.00 | 0.00 |
| PrL | prelimbic cortex | 0.20 | 0.22 | 0.33 | 0.00 | 0.11 | 0.00 |
| PT | parietal cortex | 0.07 | 0.00 | 0.00 | NA | NA | 0.00 |
| PTN | pretectal nucleus | 0.25 | 0.00 | NA | NA | 0.67 | 0.00 |
| PVH | paraventricular hypothalamic nucleus | 0.95 | 1.00 | 1.00 | 1.00 | 1.00 | 1.00 |
| PVi | periventricular hypothalamic nucleus -intermediate | 0.64 | 0.92 | 0.83 | 1.00 | 0.33 | 0.92 |
| PVpo | periventricular hypothalamic preoptic part | 0.96 | 1.00 | 1.00 | 1.00 | 1.00 | 1.00 |
| PVT | paraventricular nucleus of the thalamus | 0.67 | 1.00 | 0.89 | 0.44 | 1.00 | 0.95 |
| RCH | retrochiasmatic area | 0.76 | 0.83 | NA | 0.67 | 1.00 | 1.00 |
| RE | nucleus of reuniens | 0.44 | 0.93 | 0.50 | 0.22 | 1.00 | 0.40 |
| RH | rhomboid nucleus | 0.17 | 0.33 | NA | 0.00 | 1.00 | 0.00 |
| RS | retrosplineal area | 0.00 | 0.00 | 0.00 | 0.00 | 0.00 | 0.00 |
| rt | reticular nucleus of the thalamus | 0.13 | 0.00 | 0.00 | 0.22 | 0.00 | 0.07 |
| RTm | reticular nucleus -midbrain | 0.50 | 0.67 | 1.00 | 0.00 | 0.33 | 0.33 |
| Sc | somatosensory cortex | 0.02 | 0.00 | 0.00 | 0.00 | 0.00 | 0.06 |
| SC | superior colliculus | 0.13 | 0.00 | 0.00 | 0.00 | 0.33 | 0.00 |
| SCH | suprachiasmatic nucleus | 0.33 | 0.67 | 0.67 | 0.50 | 1.00 | 0.67 |
| scp | superior cerebelar peduncles | 0.33 | 0.00 | NA | NA | NA | NA |
| sm | stria medularis | 0.33 | 0.50 | 1.00 | 0.00 | 0.33 | 0.22 |
| SN | substantia nigra | 0.75 | 1.00 | NA | NA | 1.00 | 1.00 |
| SNR | substantia nigra reticular part | 0.67 | 1.00 | 0.33 | 0.33 | 1.00 | NA |
| SON | supraoptic nucleus | 0.85 | 1.00 | 1.00 | 1.00 | 1.00 | 1.00 |
| st | stria terminalis | 0.22 | 0.00 | NA | NA | NA | 0.00 |
| SUB | subiculum | 0.00 | 0.00 | 0.00 | NA | NA | 0.00 |
| Te | temporal cortex | 0.00 | 0.00 | NA | 0.00 | 0.00 | NA |
| TT | taenia tecta | 0.56 | 0.58 | 0.83 | 0.11 | 0.83 | 0.42 |
| Tu | tuberal nucleus | 1.00 | 1.00 | 1.00 | NA | 1.00 | 1.00 |
| V | visual cortical area 1 | 0.00 | 0.00 | 0.00 | NA | NA | 0.00 |
| V1 | visual 1 cortex | 0.00 | 0.00 | 0.00 | 0.00 | 0.00 | 0.00 |
| V2 | visual 2 cortex | 0.00 | 0.00 | NA | NA | 0.00 | NA |
| V3 | third ventricle | 0.20 | 0.00 | 0.00 | 0.00 | 0.00 | 0.00 |
| vhc | ventral hippocampal commissure | 0.08 | NA | NA | NA | NA | 0.00 |
| VLPO | ventrolateral preoptic area | 0.44 | 0.67 | 0.67 | 0.33 | 1.00 | 0.67 |
| VM | ventral medial thalamic nucleus | 0.04 | 0.00 | 0.00 | NA | 0.00 | 0.00 |
| VMH | ventromeidal hypothalamus | 0.29 | 0.67 | 0.67 | NA | 1.00 | 0.83 |
| VMPO | ventromedial preoptic area | 0.93 | 1.00 | 1.00 | 0.33 | 1.00 | 1.00 |
| VP | ventral pallidum | 0.00 | 0.00 | 0.00 | 0.00 | 0.00 | 0.00 |
| VTA | ventral tegmental area | 0.61 | 0.78 | NA | 0.00 | 0.83 | 1.00 |
| vtd | ventral tegmental decussation | 0.17 | 0.00 | NA | NA | 1.00 | NA |
| ZI | zona incerta | 0.25 | 0.20 | 0.00 | NA | 0.17 | 0.20 |
