# Supplementary material for: The central oxytocinergic system of the prairie vole
Source: Brain Struct Funct. 2024 Jul 23;229(7):1737–56. doi: 10.1007/s00429-024-02832-1 (PMC11374920; doi:10.1007/s00429-024-02832-1)
Supplement: Supplementary file 5 — Supplementary file5 (DOCX 27 KB) [file 429_2024_2832_MOESM5_ESM.docx]

**Supplemental Table 3**

Relationship between OXT fiber density and *Oxtr* transcript score from Inoue et al., 2022. OXT fiber SDI’s are pooled SDI’s across all male and female brains to account for the difference in number of sections analyzed. NA’s indicate regions that have an OXT SDI score, but no corresponding *Oxtr* score.

| **Abbreviation** | **Full Region Name** | **Classified Region** | **OXT Fibers SDI** | **Oxtr transcript score** | |
| --- | --- | --- | --- | --- | --- |
| TT | Tenia Tecta | Cerebral cortex | 0.54 | 4.00 | |
| AcbSh | Nucleus accumbens-shell | Striatum/Pallidum | 0.67 | 4.00 | |
| AcbC | Nucleus accumbens-core | Striatum/Pallidum | 0.53 | 4.00 | |
| LSd | Lateral septum-dorsal | Striatum/Pallidum | 0.67 | 3.00 | |
| BNST | Bed nucleus of the stria terminalis | Striatum/Pallidum | 0.73 | 3.17 | |
| MPOA | Medial preoptic area | Hypothalamus | 0.81 | 3.00 | |
| VMH | Ventromedial hypothalamic nucleus | Hypothalamus | 0.51 | 4.00 | |
| ARC | Arcuate hypothalamic nucleus | Hypothalamus | 0.82 | 3.00 | |
| PH | Posterior hypothalamic nucleus | Hypothalamus | 0.79 | 3.00 | |
| MM | Mammillary nucleus | Hypothalamus | 0.83 | 3.00 | |
| PVT | Paraventricular nucleus of the thalamus | Thalamus | 0.78 | 4.00 | |
| RE | Nucleus of reuniens | Thalamus | 0.53 | 3.50 | |
| GrO | Granule cell layer olfactory limb | Cerebral cortex | 0.14 | 4.00 | |
| AON | Anterior olfactory nucleus | Cerebral cortex | 0.32 | 3.00 | |
| FrA | Frontal association cortex | Cerebral cortex | 0.11 | 3.00 | |
| Cga | Cingulate area anterior | Cerebral cortex | 0.03 | 3.33 | |
| Cg | Cingulate cortex | Cerebral cortex | 0.05 | 3.00 | |
| RS | Retrosplineal area | Cerebral cortex | 0.00 | 4.00 | |
| MC | Motor cortex | Cerebral cortex | 0.01 | 3.00 | |
| AI | Agranular insular cortex | Cerebral cortex | 0.03 | 3.00 | |
| PT | Parietal cortex | Cerebral cortex | 0.03 | 2.67 | |
| PrH | Perirhinal cortex | Cerebral cortex | 0.01 | 3.00 | |
| V2 | Visual cortex | Cerebral cortex | 0.00 | 3.00 | |
| Te | Temportal cortex | Cerebral cortex | 0.00 | 3.00 | |
| OT | Olfactory tubercle | Striatum/Pallidum | 0.41 | 4.00 | |
| CA2 | Hippocampal area 2 | Cerebral cortex | 0.00 | 4.00 | |
| islm | Major island of Calleja | Striatum/Pallidum | 0.33 | 4.00 | |
| CPu | Caudate putamen | Striatum/Pallidum | 0.15 | 3.50 | |
| Amy | Amygdala | Cerebral cortex | 0.14 | 3.00 | |
| Co | Cortical amygdaloid nucleus | Cerebral cortex | 0.04 | 3.50 | |
| CM | Central medial thalamic nucleus | Thalamus | 0.42 | 4.00 | |
| VM | Ventral medial thalamic nucleus | Thalamus | 0.02 | 3.00 | |
| RH | Rhomboid nucleus | Thalamus | 0.24 | 4.00 | |
| LSi | Lateral septum-intermediate | Striatum/Pallidum | 0.67 | 2.00 | |
| LSv | Lateral septum-ventral | Striatum/Pallidum | 0.64 | 2.00 | |
| AHN | Anterior hypothalamic nucleus | Hypothalamus | 0.87 | 2.50 | |
| SCH | Suprachiasmatic nucleus | Hypothalamus | 0.51 | 2.00 | |
| VLPO | Ventrolateral preoptic nucleus | Hypothalamus | 0.55 | 1.00 | |
| SON | Supraoptic nucleus | Hypothalamus | 0.93 | 1.00 | |
| PVH | Paraventricular hypothalamic nucleus | Hypothalamus | 0.98 | 1.00 | |
| RCH | Retrochiasmatic area | Hypothalamus | 0.81 | 1.00 | |
| ME | Median eminence | Hypothalamus | 1.00 | 1.00 | |
| DM | Dorsomedial hypothalamus | Hypothalamus | 0.96 | 1.33 | |
| LH | Lateral hypothalamus | Hypothalamus | 0.99 | 2.00 | |
| VTA | Ventral tegmental area | Midbrain | 0.67 | 1.00 | |
| SN | Substantia nigra | Midbrain | 0.88 | 1.00 | |
| PAG | Periaqueductal gray | Midbrain | 0.94 | 1.25 | |
| IP | Interpeduncular nucleus | Midbrain | 0.67 | 1.00 | |
| DR | Dorsal raphe | Midbrain | 0.56 | 2.00 | |
| RTm | Reticular nucleus of the midbrain | Midbrain | 0.50 | 2.00 | |
| ORB | Orbital area | Cerebral cortex | 0.21 | 1.00 | |
| Sc | Somatosensory cortex | Cerebral cortex | 0.02 | 2.00 | |
| Pir | Piriform cortex | Cerebral cortex | 0.07 | 2.50 | |
| Au | Auditory cortex | Cerebral cortex | 0.03 | 2.00 | |
| Ent | Entorhinal cortex | Cerebral cortex | 0.00 | 1.40 | |
| V1 | Visual cortex | Cerebral cortex | 0.00 | 2.00 | |
| EPd | Endopiriform cortex- dorsal | Cerebral cortex | 0.11 | 2.00 | |
| DG | Dentate Gyrus | Cerebral cortex | 0.01 | 2.00 | |
| CA1 | Hippocampal area 1 | Cerebral cortex | 0.01 | 1.50 | |
| CA3 | Hippocampal area 3 | Cerebral cortex | 0.05 | 1.00 | |
| SUB | Subiculum | Cerebral cortex | 0.00 | 2.00 | |
| CLA | Claustrum | Cerebral cortex | 0.04 | 2.00 | |
| DB | Diagonal band of Broca | Striatum/Pallidum | 0.41 | 1.00 | |
| GP | Globus pallidus | Striatum/Pallidum | 0.31 | 1.50 | |
| rt | Reticular nucleus of the thalamus | Thalamus | 0.09 | 1.00 | |
| AM | Anteromedial nucleus of the thalamus | Thalamus | 0.05 | 1.00 | |
| AD | Anterodorsal nucleus of the thalamus | Thalamus | 0.00 | 1.00 | |
| ZI | Zona incerta | Hypothalamus | 0.21 | 1.50 | |
| IAM | Interanteromedial nucleus of the thalamus | Thalamus | 0.00 | 2.00 | |
| MHb | Medial habenular nucleus | Thalamus | 0.17 | 2.00 | |
| LHb | Lateral habenular nucleus | Thalamus | 0.17 | 1.00 | |
| LP | Lateral posterior thalamic nucleus | Thalamus | 0.00 | 1.00 | |
| PTN | Pretectal nucleus | Midbrain | 0.21 | 1.00 | |
| **Regions with no *Oxtr* score** | | |  |  |  |
| PVpo | Periventricular hypothalamic preoptic part | Hypothalamus | 0.98 | NA | |
| MEPO | Median preoptic nucleus | Hypothalamus | 0.88 | NA | |
| VMPO | Ventromedial preoptic area | Hypothalamus | 0.90 | NA | |
| AVPV | anteroventral periventricular nucleus | Hypothalamus | 0.83 | NA | |
| PVi | periventricular hypothalamic nucleus -intermediate | Hypothalamus | 0.74 | NA | |
| LPOA | lateral preoptic area | Hypothalamus | 0.70 | NA | |
| Tu | tuberal nucleus | Hypothalamus | 0.67 | NA | |
| f | fornix | Fiber tracts | 0.60 | NA | |
| ic | internal capsule | Fiber tracts | 0.55 | NA | |
| MS | medial septal nucleus | Striaum/Pallidum | 0.63 | NA | |
| SNR | Substantia nigra reticular part | Midbrain | 0.67 | NA | |
| csc | superior colliculus commmisure | Fiber tracts | 0.53 | NA | |
| LS | lateral septum | Striatum/Pallidum | 0.46 | NA | |
| opt | optic tract | Fiber tracts | 0.34 | NA | |
| on | optic nerve | Fiber tracts | 0.25 | NA | |
| vtd | ventral tegmental decussation | Fiber tracts | 0.33 | NA | |
| ac | anterior commissure | Fiber tracts | 0.31 | NA | |
| act | anterior commissure, temporal limb | Fiber tracts | 0.22 | NA | |
| fr | fasciculus retroflex | Fiber tracts | 0.25 | NA | |
| sm | stria medularis | Fiber tracts | 0.32 | NA | |
| IMD | intermediodorsal nucleus of the thalamus | Thalamus | 0.43 | NA | |
| LV | lateral ventricle | Ventricle | 0.18 | NA | |
| 3V | third ventricle | Ventricle | 0.10 | NA | |
| Amy | amygdala | Cerebral cortex | 0.33 | NA | |
| fa | corpus callosum- anterior forceps | Fiber tracts | 0.23 | NA | |
| och | optic chiasm | Fiber tracts | 0.13 | NA | |
| cpd | cerebral peduncle | Fiber tracts | 0.21 | NA | |
| IL | infralimbic cortex | Cerebral cortex | 0.24 | NA | |
| aco | anterior commissure, olfactory limb | Fiber tracts | 0.11 | NA | |
| mtt | mammillothalamic tract | Fiber tracts | 0.15 | NA | |
| SC | superior colliculus | Midbrain | 0.09 | NA | |
| st | stria terminalis | Fiber tracts | 0.15 | NA | |
| fi | fimbria | Fiber tracts | 0.05 | NA | |
| ml | medial lemniscus | Fiber tracts | 0.02 | NA | |
| LD | lateral dorsal thalamic nucleus | Thalamus | 0.05 | NA | |
| Po | posterior complex of the thalamus | Thalamus | 0.00 | NA | |
| aq | cerebral aqueduct | Ventricle | 0.37 | NA | |
| scp | superior cerebelar peduncles | Fiber tracts | 0.22 | NA | |
| MGN | medial geniculate nucleus | Thalamus | 0.15 | NA | |
| PrL | prelimbic cortex | Cerebral cortex | 0.14 | NA | |
| Hb | habenula | Thalamus | 0.11 | NA | |
| PF | parafascicular thalamic nucelus | Thalamus | 0.08 | NA | |
| vhc | ventral hippocampal commissure | Fiber tracts | 0.07 | NA | |
| AV | anteroventral nucleus of the thalamus | Thalamus | 0.04 | NA | |
| cc | corpus callosum | Fiber tracts | 0.02 | NA | |
| AI | agranular insular area | Cerebral cortex | 0.00 | NA | |
| AOB | accessory olfactory bulb | Cerebral cortex | 0.00 | NA | |
| bsc | brachium of superior colliculus | Fiber tracts | 0.00 | NA | |
| cg | cingulum bundle | Fiber tracts | 0.00 | NA | |
| dhc | dorsal hippocampal commissure | Fiber tracts | 0.00 | NA | |
| DLGN | dorsal lateral geniculate nucleus | Thalamus | 0.00 | NA | |
| ec | external capsule | Fiber tracts | 0.00 | NA | |
| emt | external medullary lamina of the thalamus | Fiber tracts | 0.00 | NA | |
| GL | glomerular layer of the olfactory bulb | Cerebral cortex | 0.00 | NA | |
| LD | lateral dorsal thalamic nucleus | Thalamus | 0.00 | NA | |
| LGN | lateral geniculate nucleus | Thalamus | 0.00 | NA | |
| lot | lateral olfactory tract | Fiber tracts | 0.00 | NA | |
| MD | mediodorsal nucleus of the thalamus | Thalamus | 0.01 | NA | |
